# The prediction of protein-protein interaction networks in rice blast fungus

**DOI:** 10.1186/1471-2164-9-519

**Published:** 2008-11-02

**Authors:** Fei He, Yan Zhang, Hao Chen, Ziding Zhang, You-Liang Peng

**Affiliations:** 1 State Key Laboratory for ArgoBiotechnology, College of Biological Sciences, China Agricultural University, Beijing 100193, PR China; 2 Department of Plant Pathology, China Agricultural University, Beijing 100193, PR China

## Abstract

**Background:**

Protein-protein interaction (PPI) maps are useful tools for investigating the cellular functions of genes. Thus far, large-scale PPI mapping projects have not been implemented for the rice blast fungus *Magnaporthe grisea*, which is responsible for the most severe rice disease. Inspired by recent advances in PPI prediction, we constructed a PPI map of this important fungus.

**Results:**

Using a well-recognized interolog approach, we have predicted 11,674 interactions among 3,017 *M. grisea *proteins. Although the scale of the constructed map covers approximately only one-fourth of the *M. grisea*'s proteome, it is the first PPI map for this crucial organism and will therefore provide new insights into the functional genomics of the rice blast fungus. Focusing on the network topology of proteins encoded by known pathogenicity genes, we have found that pathogenicity proteins tend to interact with higher numbers of proteins. The pathogenicity proteins and their interacting partners in the entire network were then used to construct a subnet called a pathogenicity network. These data may provide further clues for the study of these pathogenicity proteins. Finally, it has been established that secreted proteins in *M. grisea *interact with fewer proteins. These secreted proteins and their interacting partners were also compiled into a network of secreted proteins, which may be helpful in constructing an interactome between the rice blast fungus and rice.

**Conclusion:**

We predicted the PPIs of *M. grisea *and compiled them into a database server called MPID. It is hoped that MPID will provide new hints as to the functional genomics of this fungus. MPID is available at .

## Background

*Magnaporthe grisea *is the causal agent of rice blast disease, which occurs as outbreaks in all rice-growing regions. It is estimated that 10–30% of the annual rice harvest is lost due to this disease, which is enough to feed 60 million people [1,2]. Therefore, it is extremely important for us to better understand this fungus and to find an effective way to control it. *M. grisea *has become the principal model organism for studying the molecular basis of fungal diseases in plants because of the genetic and molecular tractability of both the fungus and rice [3].

One of the basic characteristics of biological organization is that everything in an organism can be regarded as part of a complex network [4,5]. Traditional researches rely on a single gene or protein alone and therefore do not provide a complete understanding of the biological processes. As *in vivo *elementary molecular components, proteins perform their functional roles through their interactions with one another. Thus, developing a protein-protein interaction (PPI) network can lead to a more comprehensive understanding of the cellular processes [6]. In the past few years, high-throughput methods have been implemented to identify PPIs [7-11]. Using these experimental methods, such as yeast two-hybrid screens, PPI networks for a series of model organisms were determined that allow us to understand the function of proteins at the level of systems biology. Unfortunately, none of these high-throughput methods has been applied to the rice blast fungus, despite its importance [12]. The genome sequence of *M. grisea *was released in 2005 [2], offering the first instance of the gene inventory required by a pathogenic fungus to cause plant disease. Compared with the available genomic information, the PPI data for *M. grisea *are limited. Therefore, a PPI network of *M. grisea *is urgently required to direct our further investigation of this fungus.

In parallel with the large-scale experimental determination of PPI, many PPI prediction methods were also developed. These methods are based on diverse attributes, concepts, or data types, such as interolog [13], gene expression profiles [14], gene ontology (GO) annotations [15], domain interactions [16], co-evolution [17], and structural information [18]. Some machine learning methods, such as support vector machines (SVMs), have also been used to predict PPIs [19,20].

Among the above-mentioned computational methods, the interolog approach has been widely implemented [21] and has proved to be reliable for predicting PPI from model organisms [22]. The core idea of the interolog approach is that many PPIs are conserved in different organisms [23]. Based on this approach, the first draft of a human PPI map was generated [24]. Continuously accumulated PPI data from model organisms as well as advances in detecting orthologous proteins in different organisms [25] have made the interolog method an increasingly powerful tool for constructing PPI maps for entire proteomes.

Using the interolog method, 11,674 PPIs among 3,017 *M. grisea *proteins were inferred from the experimental PPI data in different organisms. Although the predicted PPI network covers approximately only one-fourth of the *M. grisea *proteome and may still contain many false-positives, it is the first PPI network for this pathogen and will provide a framework for the future study of rice blast fungus biology.

## Results and discussion

### Generation of a *M. grisea *PPI map

With the assistance of the InParanoid algorithm [25] in identifying true orthologs between *M. grisea *and other organisms, the combined PPI data from *E. coli*, *S. cerevisae*, *C. elegans*, *D. melanogaster*, and *H. sapiens *were used to infer the PPI network of *M. grisea*. In this work, 11,674 interactions among 3,017 *M. grisea *proteins were obtained (see Additional file 1 for the full list of predicted PPIs). Approximately two-thirds of the interactions can be directly inferred from the PPI data of yeast (Table 1).

**Table 1:** The predicted protein-protein interactions

Organism	Predicted PPI
*S. cerevisae*	7,904
*C. elegans*	242
*D. melanogaster*	1,080
*H. sapiens*	2,177
*E. coli*	916

Total^a^	11,674

^a ^Considering some PPIs can be inferred from two or more organisms, the total number of predicted PPIs is 11,674.

Since the false-positive rate of the current large-scale experimental PPI data is quite high [26], the PPIs based on the interolog method will inevitably contain a large proportion of false-positives. Two strategies were utilized to increase the confidence level of the predicted data. First, we used a stringent algorithm (i.e., InParanoid) to distinguish true orthologs from out-paralogs [25]. Second, we used only the PPI data collected in the DIP [27] and HPRD [28] databases, which are manually curated and hence are of higher quality than other databases available to the community.

### Network validation

Due to the absence of large-scale experimental PPI data in *M. grisea*, we had no direct method of assessing the overall quality of the predicted network. Using the GO annotations, the domain interaction database (i.e., the iPfam database [29]), and the gene expression profiles, three computational analyses were carried out to evaluate the global quality of the predicted PPIs indirectly. The procedures involved, including the preparation of datasets and the construction of randomized networks, are detailed in the Methods section.

**Figure 1:**
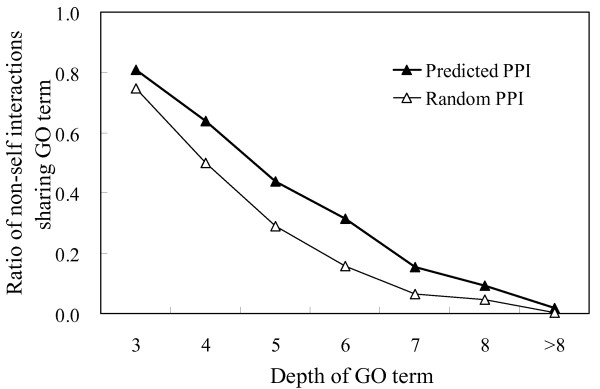
**Validation of the predicted PPI network based on shared GO annotations**. The percentage of interactions sharing GO terms at various depths in the GO hierarchy is compared with randomly generated networks of the same group of proteins. For instance, 80.8% (8,310/10,288) of the predicted PPIs share at least one GO term at depth three in the GO hierarchy, while the largest proportion in the 1,000 randomized networks is 74.6% (7,676/10,288).

The reliability of the entire network was first assessed by a method used in a previous study [24]. The main idea of this method is that two interacting proteins would be expected to have similar or related functions. Therefore, PPI datasets with high confidence should predict a greater proportion of interactions between functionally related proteins than those with low confidence. Recently, the *M. grisea *genome annotation team at North Carolina State University released the GO annotation dataset of *M. grisea*, which is based on experimental data and stringent computational approaches. The GO annotations of 7,279 proteins in *M. grisea *are available, covering 2,876 proteins and 10,288 non-self interactions in our predicted PPI network. Since a pair of interacting proteins generally have related but not identical functions, they should have some but not all of their GO annotations in common. To evaluate the network, we compared the proportion of the interactions that shared at least one GO term in any of the three GO categories in the predicted and 1,000 randomized networks. Since the GO annotations offer a hierarchical description of gene functions, in which deeper GO terms indicate more precise functions, comparisons were performed at different levels of the GO hierarchy (i.e., GO annotations at depths of 3 to 8 and more than 8). It was found that the percentage of PPI pairs sharing GO terms in the predicted PPI network was consistently higher than the largest percentage in the 1,000 randomized networks (Figure 1), suggesting that the predicted PPI network preferentially connects proteins sharing GO terms at any level of the GO hierarchy (empirical *p*-value < 0.001).

The second validation method relies on the assistance of the iPfam database. By collecting binary interacting Pfam domain pairs generated through protein three-dimensional structural data, the iPfam database is independent of the large-scale PPI data in model organisms. The iPfam database has previously been employed to predict PPIs [30], but it was used here as a reference database to validate our predicted network. The central idea for such a validation is that high-confidence PPI networks should contain a greater proportion of interactions associated with Pfam domain interacting pairs. The corresponding Pfam domains for 2,677 proteins in the predicted network were annotated, and there are 9,836 predicted PPIs among these 2,677 proteins. Of these 9,836 PPIs, 848 are associated with Pfam interacting domain pairs; however, in the 1,000 randomized networks, the average number of PPIs associated with Pfam interacting domain pairs is 42 ± 6.33. The largest number of PPIs associated with Pfam interacting domain pairs is only 62, which is significantly smaller than that of our predicted network (empirical *p*-value < 0.001).

The third method was applied to further complement the validation of the predicted PPI data. Because interacting proteins tend to have correlated gene expression profiles [31,32], this property has also been used to predict or validate protein-protein interactions [22,33]. In our predicted network, 2,874 proteins have the corresponding gene expression profiles deposited in the *M. grisea *microarray dataset from the GEO database [34]. Actually, 10,007 non-self PPIs were predicted among these 2,874 proteins. The average absolute value of the Pearson Correlation Coefficients (PCC) between the expression data of any interacting pair was 0.440. All the average absolute PCC values among the 1,000 randomized networks (0.409 ± 0.0001) are smaller than that of the predicted PPI network, which means that the predicted interacting pairs prefer to connect genes with correlated expression profiles (empirical *p*-value < 0.001).

It is important to note that the three validation methods above are somewhat indirect ways to evaluate the predicted PPIs and may still contain some drawbacks. For instance, because more than half of the GO annotations of *M. grisea *were transferred from model organisms, a certain bias inevitably exists in the first validation method. Even so, these three methods together provide convincing evidence that the confidence of the predicted PPI network is significantly higher than that of randomized networks. Therefore, we argue that the overall quality of the predicted PPIs is good.

### Validation of each predicted interaction

While the above analysis has shown the overall quality of the predicted interactions, it is also very important to evaluate the reliability for each predicted PPI. In this work two *p*-values based on GO terms and the expression correlation for each predicted interaction were proposed to assess the reliability of each predicted interaction. The underlying null model is that of randomized networks. In the randomized networks, the number of shared GO terms and the expression correlation coefficient (*i.e*., PCC) for each predicted interaction were calculated. Based on such null distributions of GO terms and expression correlation, two nominal *p*-values can be determined for each predicted interaction. More details about the calculation of *p*-values are available in the Methods section.

The calculated *p*-values allow us to determine which of the predicted PPIs are likely to be products of random processes and which are more likely to be reliable. Generally, predicted PPIs with lower *p*-values should have higher reliability. Of the 11,674 predicted PPIs, 1,757 interactions were found to have GO terms-based *p*-values ≤ 0.05, while 955 interactions were found to have expression correlation-based *p*-values ≤ 0.05. Since proteins sharing similar functions and proteins with almost identical expression profiles do not necessarily interact with each other, the GO terms and expression correlation are not really gold standards for evaluating the reliability of predicted interactions. In other words, a nominal *p*-value of 0.05 may not indicate that the predicted PPI should have a false-positive rate of 0.05.

In addition to the above two *p*-values, a prediction score *S *was assigned for each predicted interaction. Based on the Inparanoid score, the score *S *mainly reflects the orthologous relationship between the predicted interacting proteins and their corresponding experimentally validated interacting proteins (*i.e*., interologs) in model organisms. More details about the definition of *S *are addressed in the Methods section. Generally, a prediction with a higher *S *tends to be more reliable. Although we are not able to quantify the level of the score *S *that enables us to consider an interaction reliable, the score *S *can be used as a complementary measure to evaluate the reliability of a predicted interaction. In Additional file 1, we list the two nominal *p*-values and the prediction score *S *for each predicted interaction. We also annotate each interaction with its Pfam interacting domain pair (if available) and indicate from which organisms the inference came. Taken together, these measures provide an overall impression on the reliability of each predicted interaction.

### Network properties

**Figure 2:**
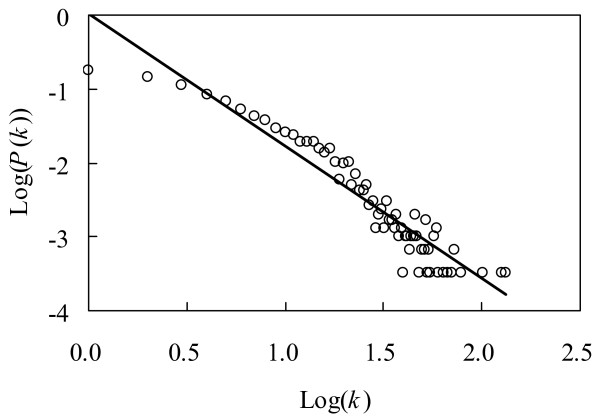
**Scale-free topology of the predicted network**. Distribution of the number of connections of nodes in the PPI network of *M. grisea *with both axes plotted on logarithmic scales.

The network properties are presented in Table 2. In our network, 55 of the 3,017 proteins have degrees higher than 40; these proteins are called hubs in the network. Some of these hubs may be the proteins encoded by essential genes in *M. grisea*. Yeast orthologs for 28 of the 55 hub proteins were reported to be essential genes (see Table S1 in Additional file 2). Since these 55 hub proteins usually perform important cellular functions, they can be a valuable resource for studying this pathogen. For instance, some of them may be selected as anti-fungus drug targets. Compared with the established PPI networks in some other organisms, our network generally has a larger diameter and a smaller clustering coefficient [33], implying that our network is somewhat loosely connected; however, compared with 1,000 randomized PPI networks with the same degree distribution (Table 2), our network has a larger clustering coefficient, which means local cohesiveness exists in the predicted PPI network, and clusters representing biological complexes or pathways may be detected [35]. Considering that the current network is far from complete, these parameters reflect only the limited PPI data in our network. As the availability of experimental PPI data increases in the future, the detailed parameters of these network properties will be changed. Even so, the so-called 'scale-free' topology can be more or less observed in our network (Figure 2). Scale-free networks typically have many nodes with few links and only a few highly connected ones, which have been frequently observed in the PPI networks of other organisms. In contrast to a random network, in which the connectivity distribution obeys a Poisson distribution, in a scale-free network the probability *P*(*k*) of nodes having *k *edges decays as a power law, *P*(*k*) ≈ *k*^-γ^. We plotted the connectivity distribution on a double logarithmic scale to identify the most reliable linear fit for the data, characteristic of a scale-free topology (Figure 2). The established network in *M. grisea *is approximately characterized by a power law, where *P*(*k*) ≈ *k*^-1.79 ^(*R*^2 ^= 0.90).

**Table 2:** Topological properties of the PPI network in *M. grisea*

	Predicted network	Randomized network ^a^
Average Degree	7.74	7.74
Diameter	9.00	6.00. ± 0.032
Average Distance	4.03	3.66. ± 0.003
Clustering Coefficient	0.11	0.01. ± 0.001

^a ^The randomized network was generated by keeping the degree distribution constant and randomizing the number of edges of the predicted network. Briefly, we first randomly allocated the number of edges for each node according to the degree distribution of the predicted network. Subsequently, we randomly picked a pair of nodes to make an edge, then decrease the degree for both nodes by one until the expected edges has been assigned to each node (i.e., the remained degree for each node decreases to 0). This process was repeated 1,000 times, and the average values and standard deviations of the corresponding network properties are shown.

### Pathogenicity genes in the network

Pathogenicity genes play key roles during the fungal infection process. A fungus will lose all or part of its pathogenicity when a pathogenicity gene is knocked out. Previous work has demonstrated that the diverse functions of pathogenicity genes have no preferential enrichment in any GO category, in comparison with the analysis of all genes in *M. grisea *[36]. Of the 100 pathogenicity genes collected from the PHI-base website and the recently published work of Lee and co-workers [36,37], only 32 pathogenicity proteins can be found in the established network (see Additional file 2 for more details). The average degree of these 32 pathogenicity proteins is 10.25, which is higher than that of the whole network (7.74, *p*-value = 0.150). The pathogenicity genes uncovered by Lee and co-workers were based on a high-throughput phenotype screening, and they may need further experimental validation. For comparison, the pathogenicity genes deposited in the PHI-base were verified by individual experiments and share a higher confidence level. Considering only the pathogenicity proteins from the PHI-base, the average degree is 11.74, which is much higher than that of the whole network (*p*-value = 0.077). Therefore, it seems that the pathogenicity proteins tend to have higher degrees.

In the interolog method, the number of interacting partners predicted for a *M. grisea *protein is related to the number of orthologs this protein has in the five model organisms. We analyzed the number of orthologs of all 3,017 proteins in the predicted network and found that the average number of orthologs of the 32 pathogenicity proteins is 4.06, which is higher than that of all 3,017 proteins (3.59, *p*-value = 0.305). We further observed that the average number of orthologs of pathogenicity proteins from the PHI-base is 4.47, which is higher than the average number of all 3,017 proteins (*p*-value = 0.141). Although neither of these facts is statistically significant, we are unable to fully rule out the argument that the higher connectivity of pathogenicity proteins may be a result of the larger number of orthologs they have in the five model organisms.

Researchers investigating an interactome have frequently used the number of interacting partners of a protein as an important parameter reflecting the protein's cellular function. For instance, there is increasing evidence for a correlation between the evolutionary conservation of a protein and the number of its interacting partners [38,39]. It is well accepted that proteins that participate in more interactions are phenotypically more important for the organism [35]. It has also been observed that the toxicity-modulating proteins in *S. cerevisiae *are involved in a larger number of interactions [40]. Additionally, human cancer proteins have been reported to have far more interacting partners than other human proteins [41]. It is shown here that the increased connectivity of pathogenicity proteins may indicate their special biological roles in the pathogen.

**Figure 3:**
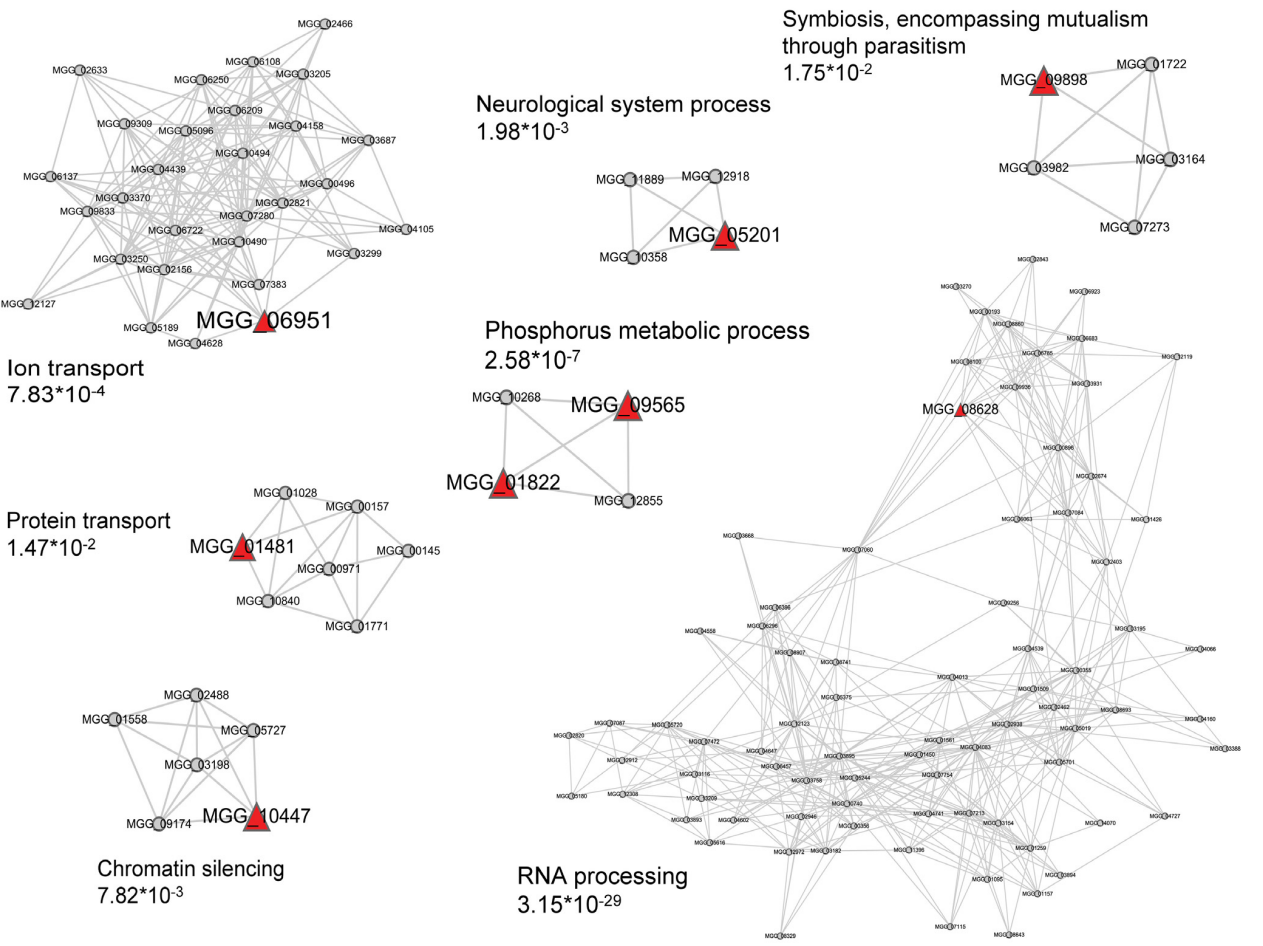
**Clusters or communities containing pathogenicity genes (proteins)**. Those nodes represented by red triangles are pathogenicity genes. The description alongside each cluster is the enriched GO term identified by the Fisher exact test followed by the FDR correction, and the corresponding corrected *p*-value is also listed. The clusters were drawn using Cytoscape2.5.1 [57]. A larger version of this figure is available in Additional file 3.

In order to understand the cellular function of a protein on a systems level, it is increasingly important to study the network or functional module involved. It has been proved that clustering methods are good at identifying PPIs that take place within the same pathways or complexes [42,43]. Using identified clusters (also called functional modules) in a network, we can predict the functions of proteins within the clusters [44,45]. Using the *k*-clique clustering method [46], we detected some clusters with pathogenicity genes (Figure 3 and Additional file 3). Recent studies have shown that human disease genes can be predicted based on the human PPI data, because mutations in different members of a protein complex can often lead to similar diseases [47,48]. Likewise, mutations in different members of a protein complex may lead to similar pathogenicity phenotypes. Therefore, new pathogenicity genes are likely to be found within these clusters.

The identified clusters in Figure 3 allow us a glimpse of the functional diversity of pathogenicity genes. The GO enrichments in these identified clusters include ion transport, chromatin silencing, RNA processing, and phosphorus metabolic processes (Figure 3). The potential biological impact of these clusters is exemplified in one cluster with a GO enrichment of phosphorus metabolic processes. Interestingly, in this cluster two pathogenicity proteins, PMK1(MGG_09565) and OSM1(MGG_01822), were predicted to have an interaction and were also found to be tightly connected with PBS2(MGG_10268) and MST11(MGG_12855). All four of these proteins are protein kinases. It has been established that the MST11-MST7-PMK1 cascade regulates the appressorium formation and infectious growth of *M. grisea*, and this pathway might have crosstalk with the other two MAPK cascades (MPS1 and OSM1 cascades) [49]. Unfortunately, the molecular mechanisms involved in the interactions among the three pathways are not yet clear. The cluster presented here may provide some hints on the crosstalk between the MST11-MST7-PMK1 and OSM1 cascades.

The pathogenicity of *M. grisea *is complicated, and it is necessary to consider it a network [50]. Furthermore, the pathogenicity proteins in the network together with their interacting partners are compiled into a subnet in which most of the pathogenicity proteins are connected into one major component (see Additional file 4). This so-called pathogenicity protein network may provide a clue to understanding the pathogenicity of the rice blast fungus at a systems level. Taken together, the clusters identified above and the pathogenicity protein network will be helpful for the study of fungal pathogenesis and the identification of new pathogenicity genes in the rice blast fungus.

### Secreted proteins in the network

The secreted proteome is a crucial component reflecting the ability of fungi to perceive and respond to the environment. The genome of *M. grisea *contains a large number of secreted proteins, which play important roles in the attachment to and the colonization within plant tissues [2]. Of the 1,452 secreted proteins predicted by the SignalP program [51], 105 are included in our network (Table S3 in Additional file 2). All of the secreted proteins have degrees less than 40 and the average degree is only 4.81, which is significantly smaller than that of the whole network (*p*-value = 3.30 × 10^-5^). As secreted proteins are secreted out of cells, it is reasonable to expect them to have low connectivity in the network.

Some of these secreted proteins may be secreted into the rice tissue and interact with rice proteins [50]. It can be hypothesized that this group of secreted proteins have a much lower degree in the *M. grisea *protein network. We further analyzed the 105 predicted secreted proteins using WoLFPSORT [52] and found that 28 proteins were not predicted to be located outside of the cell of *M. grisea*. In other words, these 28 proteins may be secreted into host cells and interact with host proteins (see Table S4 in Additional File 2 for more detailed information). The average degree of these 28 proteins in the established network is only 4.07, in accordance with our hypothesis (*p*-value = 5.22 × 10^-5^). Furthermore, these 105 secreted proteins, together with their interacting partners, were compiled into a subnet of secreted proteins in *M. grisea *(see Additional file 5) to facilitate further study of the host-pathogen interactions between *M. grisea *and rice.

The secreted proteins' orthologs in the five model organisms were also counted. The average number of orthologs of the 105 secreted proteins in our network was observed to be 2.85, which is smaller than that of all the proteins in the predicted network (*p*-value = 1.49 × 10^-4^). Furthermore, we found that the average number of orthologs of the 28 secreted proteins that may interact with rice proteins is 3.29, which is also slightly smaller than that of all the proteins in the predicted network (*p*-value = 0.545). Therefore, we cannot fully rule out the argument that the lower connectivity of secreted proteins may be a result of the generally smaller number of orthologs they have in the five model organisms.

### Utility

**Figure 4:**
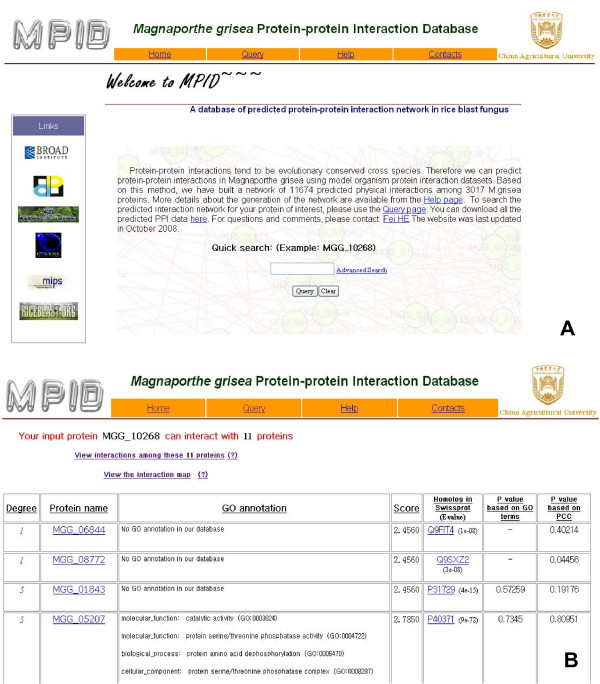
**The snapshot of the MPID website**. (A) The homepage of MPID. (B) The search result provided by MPID.

The predicted data can be accessed on the MPID website . Users can input a protein's BROAD accession number, and a table listing the predicted interacting partners of the query protein is returned (Figure 4A and 4B). This table provides the nominal *p*-values, the prediction scores, the corresponding GO annotations, and the Swiss-Prot homologs for interacting proteins. Users can also view an image generated by Graphviz  of the interaction subnet around the input protein. MPID also allows users to input a group of proteins to ascertain the interactions among them.

## Conclusion

Using a well-recognized interolog approach, we compiled a draft map of the PPI network in *M. grisea*, which can be characterized as a "scale-free" network. The reliability of our network has been clearly demonstrated by the results of three validation methods. For each predicted interaction within the network, two nominal *p*-values based on GO terms and the expression correlation were proposed to assess its reliability. Although the established network is far from complete and certainly contains many false positives, we hope it can provide new insights into the rice blast fungus.

We have found that the pathogenicity proteins encoded by the pathogenicity genes tend to have a higher average degree than that of the whole network, reflecting their special biological roles in the organism. We also observed that secreted proteins interact with fewer proteins. Recently, a high-throughput analysis of the rice blast fungus genome was initiated, an indication that fungal genomics goes industrial [53]. In this context, the availability of this network will be helpful for future high-throughput PPI mapping projects. For instance, it may play an important role in choosing bait and prey in yeast two-hybrid experiments.

## Methods

### Building a protein-protein interaction network for *M. grisea*

The protein sequences of *M. grisea *were obtained from the *M. grisea *database (release 5) maintained by the BROAD institute [2], which contains a total of 12,832 sequences. The experimentally identified PPI maps from five model organisms (*E. coli*, *S. cerevisae*, *C. elegans*, *D. melanogaster*, and *H. sapiens*) were used to infer the PPI network of *M. grisea*. The corresponding PPI data were downloaded from DIP and HPRD (see Additional file 6 for more details about the data used in this paper).

The orthologs of *M. grisea *proteins in *E. coli*, *S. cerevisae*, *C. elegans*, *D. melanogaster*, and *H. sapiens *were identified using InParanoid with the default settings. To find orthologs for a query sequence, InParanoid first identified all the potential orthologs in the target organism by pair-wise similarity searching and then clustered these potential orthologs into groups of likely co-orthologs. Here, we selected only the main orthologs to construct the PPI network. For any two proteins in *M. grisea*, an interaction was predicted if their orthologs in five different model organisms have at least one experimentally verified interaction. For example, if *A *and *B *are two proteins in *M. grisea *whose corresponding orthologous proteins in *S. cerevisae *(*A*' and *B*') were reported to have one interaction, then we predicted that *A *may interact with *B*.

Moreover, we assigned a prediction score *S *for each PPI by using a strategy similar to that proposed by Jonsson and Bates [41]. Given a predicted interaction pair *A *and *B*, the prediction score *S *is defined as

(1) $$S=\sum_{i=1}^{N}\log[IS({A^{\prime }}_{i},A)\times IS({B^{\prime }}_{i},B)]$$

where ${A^{\prime }}_{i}$ and ${B^{\prime }}_{i}$ are the corresponding orthologs of *A *and *B *that were reported to have an interaction in one model organism (i.e., the protein pair ${A^{\prime }}_{i}$ and ${B^{\prime }}_{i}$ is called an interolog of protein pair *A *and *B*); *IS*(${A^{\prime }}_{i}$, *A*) is the InParanoid score between ${A^{\prime }}_{i}$ and *A*, while *IS*(${B^{\prime }}_{i}$, *B*) is the InParanoid score between ${B^{\prime }}_{i}$ and *B*; *N *is the total number of interologs of protein pair *A *and *B *identified in the five model organisms.

### Network validation

Three computational experiments were designed to validate the quality of our predicted PPI network. In the first computational experiment, the recently released GO annotations of *M. grisea *were downloaded from the GO website [54]. 7,279 proteins in the *M. grisea *proteome can be annotated by specific GO terms, of which 2,876 are included in our predicted PPI network. There are 10,288 predicted non-self interactions among the 2,876 proteins. For comparison, 1,000 randomized PPI networks were constructed. In each randomized network, 10,288 non-self pairs were randomly selected from the 2,876 proteins. We then compared the proportion of PPIs sharing at least one GO term in the predicted and randomized networks. We calculated the proportion of PPIs sharing a GO term at depths of 3 to 8 and more than 8 in the GO hierarchy to avoid this result just applied to quite general GO terms. Note that the GO terms from three categories were taken into account in this analysis. For the GO terms with more than one path to the GO root, we defined the depth of the corresponding GO terms as the shortest path length.

In the second validation method, the Pfam domain annotations for the proteins in our predicted PPI network were generated by employing the locally installed Hmmer-2.3.2 and Pfam database (Pfam_ls, release 22.0) [55]. For each query protein in the predicted PPI network, a Pfam search was performed with the default settings. Using a 0.01 *E*-value cut-off, we were able to assign Pfam domain annotations for 2,677 proteins, covering 9,836 predicted PPIs. Moreover, we counted the number of PPIs associated with Pfam domain interacting pairs in the current iPfam database (version 21.0) [29]. To facilitate comparison, 1,000 randomized PPI networks were also constructed, in each of which 9,836 protein pairs were randomly selected from the 2,677 proteins. We then counted how many randomly generated pairs could be associated with Pfam interacting domain pairs in each randomized network. Finally, the proportion of PPIs associated with Pfam domain interacting pairs in the predicted and randomized networks was used to assess the quality of our predicted network.

To perform the third validation, we first downloaded a set of *M. grisea *microarray data from the GEO database (Accession: GSE1945) [34], which detected differential gene expression during the germination and appressorium formation of the rice blast fungus. 2,874 proteins in our predicted network were found to have the available gene expression profiles in the microarray data. We then computed the average absolute value of PCC between the expression data of any interacting pairs in these 2,874 proteins. A total of 10,007 non-self PPIs were predicted among these 2,874 proteins. For comparison, 1,000 randomized networks were generated, in each of which 10,007 non-self interacting pairs were selected among these 2,874 proteins. To assess the quality of our predicted PPI network, the average absolute value of PCCs in our predicted network and the 1,000 randomized networks were compared.

### Validation of each predicted interaction

Two *p*-values based on GO terms and the expression correlation were proposed to assess the reliability of each predicted interaction, by comparing it with the set of interactions within randomized networks. Using the 1000 randomized networks we generated for the GO annotations-based network validation, we calculated the number of shared GO terms for each pair of nodes. Here only GO terms at depths ≥ 3 were taken into account. We then created a histogram of the corresponding enrichment of the number of shared GO terms. This null distribution of the number of shared GO terms was used to estimate a nominal *p*-value for each predicted interaction. For instance, if a predicted PPI shares *m *GO terms, the corresponding percentage of PPIs sharing *m *or more GO terms in the null distribution is defined to be the GO terms-based *p*-value of the predicted interaction. The nominal *p*-value based on expression correlation can likewise be estimated. Using the 1,000 randomized networks we generated for the expression correlation-based network validation, we also obtained the null distribution of the absolute PCC values, which was used to estimate the nominal *p*-value for each predicted PPI. For instance, if a predicted PPI has an absolute PCC of α, the corresponding percentage of PPIs with absolute PCC values ≥ α in the null distribution is assigned as the expression correlation-based *p-*value of the predicted PPI.

### Network analysis

The average degree, clustering coefficient, characteristic path length, and diameter were calculated to characterize this newly established PPI network. The degree (*i.e*., connectivity) of a node is the number of nodes that are directly linked to it. The average degree is the average of the degrees of all the nodes. The clustering coefficient of a node is the ratio between the number of existing links between its neighbors and the maximum possible number of links between them. The clustering coefficient of a network, which can be used to investigate its local cohesiveness, is the average of the clustering coefficients of all the nodes. The characteristic path length is the average minimum distance between any two nodes, indicating how closely nodes are connected within the network. The diameter of a network is the longest graph-theoretical distance between any two nodes in the network.

### Clustering of network

Some proteins in the PPI network appear as clusters, in which the nodes are more highly connected to one another compared to the rest of the network [35,56]. To identify meaningful clusters of the established network, the CFinder program [46] was employed. This method first located maximal complete subgraphs (*k*-cliques) in the network. "Communities" were then detected by carrying out standard component analysis of the clique-clique overlap. The clustering method can be applied at different *k*-values, with higher *k*-values generating protein communities with higher degrees of interconnection. In this study, a *k*-value of 4 was selected. In each identified cluster, the GO enrichment in the GO category of the biological process was determined by using the Fisher exact test followed by the False Discovery Rate (FDR) correction. The corrected *p*-value was calculated for each GO term at a depth of 4 in the GO hierarchy. The most significantly over-represented GO term was assigned to each cluster.

### Proteins encoded by pathogenicity genes of *M. grisea*

The pathogenicity genes were queried from the PHI-base (Version 2.3.1) [37] and a recent publication of Lee et al. [36]. There are currently 42 pathogenicity genes of *M. grisea *in the PHI-base website. Among the 202 new pathogenicity loci uncovered by Lee and co-workers, we used only 61 loci, which are exactly located on open reading frames. Considering that there are three overlapping genes in the above resources, a total of 100 pathogenicity genes were obtained for further analysis.

### Prediction of secreted proteins in *M. grisea*

The secreted proteins in the *M. grisea *proteome were predicted by SignalP3.0[51], which detects the presence and location of signal peptide cleavage sites in protein sequences based on Neural Network and Hidden Markov Model algorithms. To perform a prediction, only the first 70 residues in the N-terminal of a query protein were processed and the other default parameters of SignalP3.0 were used. We considered as secreted proteins only those that were consistently predicted by both algorithms. Moreover, WoLFPSORT[52], a predictor based on known sorting signal motifs and some sequence features, such as amino acid composition, was employed to identify the subcellular location of the secreted proteins.

## Authors' contributions

FH wrote programs, developed the MPID database, and drafted the manuscript. HC participated in the analysis of data. YZ, ZZ, and YLP conceived the study. YZ helped draft the manuscript. YLP provided useful suggestions to explain the results. ZZ directed the research and critically revised the manuscript. All the authors have read and approved the final manuscript.

## Supplementary Material

Additional file 1 **The predicted PPIs in *M. grisea*.** This file contains all the predicted protein-protein interaction pairs in *M. grisea*. For each predicted PPI pair, we list the corresponding BROAD accession numbers for two proteins, the two nominal *p*-values based on GO terms and the expression correlation, the prediction score *S*, the annotated Pfam interacting domain pair (if available), and the organisms that the inference came from. Click here for file

Additional file 2 **The information of hub proteins, pathogenicity proteins, and secreted proteins.** This file contains Tables S1, S2, S3, and S4. Tables S1, S2, and S3 show the detailed information about the 55 hub proteins, 32 pathogenicity proteins, and 105 secreted proteins in *M. grisea*. Table S4 shows the GO annotations of 28 secreted *M. grisea *proteins that may interact with rice proteins. Click here for file

Additional file 3 **Clusters or communities containing pathogenicity genes (proteins).** This pdf file contains a larger version of Figure 3. Click here for file

Additional file 4 **The network of pathogenicity proteins.** This file contains a network graph showing 32 pathogenicity proteins and their interacting partners. Click here for file

Additional file 5 **The network of secreted proteins. **This file contains a network graph showing 105 secreted proteins and their interaction partners. Click here for file

Additional file 6 **The sources of proteome and PPI data of the model organisms. **This file contains a table (i.e., Table S5) showing the websites and versions of the proteome and PPI data of the model organisms used in this paper. Click here for file
