# Supplementary material for: The prediction of protein-protein interaction networks in rice blast fungus
Source: BMC Genomics. 2008 Nov 2;9:519. doi: 10.1186/1471-2164-9-519 (PMC2601049; doi:10.1186/1471-2164-9-519)
Supplement: Additional file 2 — The information of hub proteins, pathogenicity proteins, and secreted proteins. This file contains Tables S1, S2, S3, and S4. Tables S1, S2, and S3 show the detailed information about the 55 hub proteins, 32 pathogenicity proteins, and 105 secreted proteins in M. grisea. Table S4 shows the GO annotations of 28 secreted M. grisea proteins that may interact with rice proteins. [file 1471-2164-9-519-S2.doc]

**Table S1. The hubs in the network^a,b^**

| **Protein** | **Degree** | **PFAM domain** | **Description** | **E value** | **Number** | **Yeast ortholog^c,d^** |
| --- | --- | --- | --- | --- | --- | --- |
| MGG_00193 | 40 | PF00773 | RNB domain | 7.90E-131 | 1 | YOL021C(Essential) |
| MGG_00355 | 41 | PF01423 | LSM domain | 2.70E-19 | 1 | YBL026W(Essential) |
| MGG_05857 | 41 | PF00850 | Histone deacetylase domain | 4.50E-143 | 1 | YNL330C(Non essential) |
| MGG_11350 | 41 | PF08423 | Rad51 | 6.40E-206 | 1 | YER095W(Non essential) |
| MGG_02511 | 42 | PF00297 | Ribosomal protein L3 | 2.40E-55 | 1 | YGR220C(Non essential) |
| MGG_03270 | 42 | PF03813 | Nrap protein | 2.00E-150 | 1 | YGR090W(Essential) |
| MGG_04008 | 42 | PF00573 | Ribosomal protein L4/L1 family | 4.20E-08 | 1 | YML025C(Essential) |
| MGG_01292 | 43 | PF01189 | NOL1/NOP2/sun family | 2.20E-136 | 1 | YNL061W(Essential) |
| MGG_03537 | 43 | PF02985 | HEAT repeat | 8.60E-23 | 5 | YMR308C(Essential) |
| MGG_00908 | 44 | PF07728 | ATPase family associated with various cellular activities (AAA) | 0.00028 | 1 | YDR394W(Essential) |
| MGG_03520 | 44 | No hit. | - | - | - | YLR074C(Non essential) |
| MGG_08907 | 44 | PF00076 | RNA recognition motif. (a.k.a. RRM, RBD, or RNP domain) | 7.20E-21 | 1 | YIL061C(Essential) |
| MGG_03071 | 45 | PF05669 | SOH1 | 3.40E-26 | 1 | YGL127C(Non essential) |
| MGG_03165 | 45 | PF00118 | TCP-1/cpn60 chaperonin family | 2.90E-179 | 1 | YLR259C(Essential) |
| MGG_09208 | 45 | PF03810 | Importin-beta N-terminal domain | 3.90E-07 | 1 | YBR017C(Non essential) |
| MGG_03668 | 46 | PF03810 | Importin-beta N-terminal domain | 0.00013 | 1 | YLR347C(Essential) |
| MGG_03982 | 46 | PF00022 | Actin | 2.20E-123 | 1 | YFL039C(Essential) |
| MGG_07280 | 46 | PF01151 | GNS1/SUR4 family | 1.20E-130 | 1 | YLR372W(Non essential) |
| MGG_08162 | 46 | PF01926 | GTPase of unknown function | 0.00011 | 1 | YOR187W(Non essential) |
| MGG_08878 | 46 | PF00352 | Transcription factor TFIID (or TATA-binding protein, TBP) | 1.30E-87 | 2 | YER148W(Essential) |
| MGG_09313 | 46 | PF00400 | WD domain, G-beta repeat | 5.10E-27 | 4 | YMR049C(Essential) |
| MGG_02730 | 47 | PF00076 | RNA recognition motif. (a.k.a. RRM, RBD, or RNP domain) | 4.40E-11 | 1 | YDL213C(Non essential) |
| MGG_02829 | 47 | PF07714 | Protein tyrosine kinase | 3.10E-05 | 1 | YPL204W(Essential) |
| MGG_06099 | 47 | PF00149 | Calcineurin-like phosphoesterase | 1.60E-41 | 1 | YDL188C(Non essential) |
| MGG_05096 | 48 | PF00106 | short chain dehydrogenase | 3.20E-13 | 1 | YBR159W(Non essential) |
| MGG_06936 | 50 | PF02798 | Glutathione S-transferase, N-terminal domain | 4.80E-09 | 1 | YKL081W(Non essential) |
| MGG_07465 | 50 | PF00400 | WD domain, G-beta repeat | 2.70E-59 | 10 | YCR057C(Essential) |
| MGG_01671 | 51 | PF01912 | eIF-6 family | 3.10E-118 | 1 | YPR016C(Essential) |
| MGG_11497 | 51 | PF07653 | Variant SH3 domain | 9.30E-11 | 1 | YDR388W(Non essential) |
| MGG_01160 | 52 | PF00125 | Core histone H2A/H2B/H3/H4 | 2.50E-18 | 1 | YNL030W(Non essential) |
| MGG_06293 | 52 | PF00125 | Core histone H2A/H2B/H3/H4 | 2.50E-18 | 1 | YNL030W(Non essential) |
| MGG_06884 | 52 | PF00036 | EF hand | 1.70E-46 | 4 | YBR109C(Essential) |
| MGG_10504 | 52 | PF00400 | WD domain, G-beta repeat | 6.20E-56 | 6 | YGL137W(Essential) |
| MGG_11794 | 52 | PF02826 | D-isomer specific 2-hydroxyacid dehydrogenase, NAD binding domain | 2.70E-34 | 1 | YER081W(Non essential) |
| MGG_09284 | 53 | PF01193 | RNA polymerase Rpb3/Rpb11 dimerisation domain | 2.60E-18 | 1 | YPR110C(Essential) |
| MGG_04400 | 54 | PF00271 | Helicase conserved C-terminal domain | 1.10E-35 | 1 | YKR059W(Non essential) |
| MGG_12636 | 54 | No hit. | - | - | - | YER022W(Essential) |
| MGG_04191 | 55 | PF02491 | Cell division protein FtsA | 0.0085 | 1 | YJR045C(Essential) |
| MGG_03696 | 57 | PF07714 | Protein tyrosine kinase | 9.70E-08 | 1 | YOR061W(Non essential) |
| MGG_07007 | 57 | PF01992 | ATP synthase (C/AC39) subunit | 5.30E-169 | 1 | YLR447C(Non essential) |
| MGG_07213 | 57 | PF00515 | Tetratricopeptide repeat | 1.80E-07 | 3 | YBR055C(Essential) |
| MGG_01250 | 59 | No hit. | - | - | - | YLR295C(Non essential) |
| MGG_06209 | 59 | No hit. | - | - | - | YBR106W(Non essential) |
| MGG_07332 | 59 | PF07653 | Variant SH3 domain | 5.40E-05 | 2 | YHR114W(Non essential) |
| MGG_11251 | 59 | No hit. | - | - | - | YLR295C(Non essential) |
| MGG_06995 | 60 | PF00333 | Ribosomal protein S5, N-terminal domain | 2.20E-09 | 1 | YBR251W(Non essential) |
| MGG_04331 | 64 | PF00318 | Ribosomal protein S2 | 9.10E-43 | 1 | YHL004W(Non essential) |
| MGG_10157 | 67 | PF08785 | Ku C terminal domain like | 3.00E-08 | 1 | YMR106C(Non essential) |
| MGG_04652 | 70 | PF05001 | RNA polymerase Rpb1 C-terminal repeat | 1.80E-28 | 11 | YDL140C(Essential) |
| MGG_04617 | 73 | PF04096 | Nucleoporin autopeptidase | 3.00E-78 | 1 | YMR047C(Essential) |
| MGG_05370 | 73 | PF08271 | TFIIB zinc-binding | 1.50E-14 | 1 | YPR086W(Essential) |
| MGG_04862 | 79 | PF00071 | Ras family | 6.30E-14 | 1 | YML064C(Essential) |
| MGG_01362 | 101 | PF07714 | Protein tyrosine kinase | 1.70E-14 | 1 | YBR160W(Essential) |
| MGG_07060 | 127 | PF03130 | PBS lyase HEAT-like repeat | 0.00013 | 3 | YNL189W(Essential) |
| MGG_06318 | 132 | PF00076 | RNA recognition motif. (a.k.a. RRM, RBD, or RNP domain) | 7.80E-13 | 1 | YJR091C(Non essential) |

^a^ Those proteins have degrees ≥ 40 were assigned as hubs in our network. ^b^ The Pfam domain annotations of those proteins with *E*-value < 0.01 are provided. The name and brief description of the Pfam domain and the number of this Pfam domain it contains are provided. The Pfam domain annotation was carried out by using Hmmer-2.3.2 and Pfam_ls(release 22.0). ^c^ The yeast ortholog was identified by the InParanoid algorithm. ^d^The yeast essential gene information was acquired from http://www-sequence.stanford.edu/group/yeast_deletion_project/

**Table S2 The 32 pathogenicity proteins in the network ^a,b^,**

| **Protein** | **Degree** | **PFAM domain** | **Description** | **E value** | **Number** | **Source** |
| --- | --- | --- | --- | --- | --- | --- |
| MGG_04582 | 1 | No hit. | - | - | - | Jeon,J. and S.Y.Park, et al |
| MGG_06368 | 1 | PF07714 | Protein tyrosine kinase | 8.40E-15 | 1 | PHI-base:CPKA(PHI:36 Identity:99.81) |
| MGG_12958 | 1 | PF00096 | Zinc finger, C2H2 type | 1.90E-17 | 2 | PHI-base:MST12(PHI:268 Identity:99.72) |
| MGG_03459 | 2 | PF02893 | GRAM domain | 3.40E-17 | 1 | PHI-base:PTH8(PHI:122 Identity:98.36) |
| MGG_00529 | 3 | PF00004 | ATPase family associated with various cellular activities (AAA) | 4.00E-83 | 2 | PHI-base:PEX6(PHI:593 Identity:100.00) |
| MGG_02986 | 3 | PF03104 | DNA polymerase family B, exonuclease domain | 0.0002 | 1 | Jeon,J. and S.Y.Park, et al |
| MGG_03284 | 3 | No hit. | - | - | - | Jeon,J. and S.Y.Park, et al |
| MGG_04895 | 3 | PF00463 | Isocitrate lyase family | 0 | 1 | PHI-base:ICL1(PHI:305 Identity:100.00) |
| MGG_07528 | 3 | PF00475 | Imidazoleglycerol-phosphate dehydratase | 2.10E-95 | 1 | PHI-base:PTH3(PHI:121 Identity:99.54) |
| MGG_09471 | 3 | PF07492 | Neutral trehalase Ca2+ binding domain | 8.00E-14 | 1 | PHI-base:PTH9(PHI:123 Identity:99.86) |
| MGG_01818 | 4 | PF00503 | G-protein alpha subunit | 8.80E-181 | 1 | PHI-base:MAGA(PHI:82 Identity:99.72) |
| MGG_03530 | 4 | PF08238 | Sel1 repeat | 3.00E-35 | 7 | Jeon,J. and S.Y.Park, et al |
| MGG_04137 | 4 | PF08513 | LisH | 4.50E-08 | 1 | Jeon,J. and S.Y.Park, et al |
| MGG_07015 | 5 | No hit. | - | - | - | Jeon,J. and S.Y.Park, et al |
| MGG_08561 | 5 | PF00097 | Zinc finger, C3HC4 type (RING finger) | 1.70E-05 | 1 | Jeon,J. and S.Y.Park, et al |
| MGG_02423 | 6 | PF00810 | ER lumen protein retaining receptor | 3.10E-116 | 1 | Jeon,J. and S.Y.Park, et al |
| MGG_06320 | 7 | PF00169 | PH domain | 2.60E-10 | 1 | PHI-base:CHM1(PHI:519 Identity:100.00) |
| MGG_09250 | 8 | PF02668 | Taurine catabolism dioxygenase TauD, TfdA family | 4.70E-19 | 1 | Jeon,J. and S.Y.Park, et al |
| MGG_01721 | 10 | PF00755 | Choline/Carnitine o-acyltransferase | 2.50E-214 | 1 | PHI-base:CrAT1(PHI:594 Identity:97.92) |
| MGG_09898 | 10 | PF00788 | Ras association (RalGDS/AF-6) domain | 4.60E-11 | 1 | PHI-base:MAC1(PHI:81 Identity:99.91) |
| MGG_00365 | 12 | PF00025 | ADP-ribosylation factor family | 0.0051 | 1 | PHI-base:MAGB(PHI:83 Identity:99.72) |
| MGG_07335 | 12 | PF00027 | Cyclic nucleotide-binding domain | 7.70E-42 | 2 | PHI-base:SUM1(PHI:128 Identity:100.00) |
| MGG_08628 | 12 | No hit. | - | - | - | Jeon,J. and S.Y.Park, et al |
| MGG_06951 | 17 | PF01435 | Peptidase family M48 | 1.30E-43 | 1 | Jeon,J. and S.Y.Park, et al |
| MGG_01481 | 18 | PF00400 | WD domain, G-beta repeat | 2.60E-22 | 4 | Jeon,J. and S.Y.Park, et al |
| MGG_00383 | 19 | PF00438 | S-adenosylmethionine synthetase, N-terminal domain | 8.20E-59 | 1 | Jeon,J. and S.Y.Park, et al |
| MGG_05201 | 22 | PF00400 | WD domain, G-beta repeat | 9.10E-59 | 7 | PHI-base:MGB1(PHI:311 Identity:99.44) |
| MGG_01822 | 23 | PF07714 | Protein tyrosine kinase | 9.70E-14 | 1 | PHI-base:OSM1(PHI:153 Identity:100.00) |
| MGG_03860 | 23 | PF00982 | Glycosyltransferase family 20 | 4.00E-293 | 1 | PHI-base:TPS1(PHI:322 Identity:100.00) |
| MGG_04943 | 27 | PF07714 | Protein tyrosine kinase | 8.20E-13 | 1 | PHI-base:MPS1(PHI:113 Identity:100.00) |
| MGG_10447 | 28 | PF00160 | Cyclophilin type peptidyl-prolyl cis-trans isomerase/CLD | 1.80E-97 | 1 | PHI-base:CYP1(PHI:249 Identity:100.00) |
| MGG_09565 | 29 | PF07714 | Protein tyrosine kinase | 5.40E-14 | 1 | PHI-base:PMK1(PHI:690 Identity:99.72) |

^a^ The average degree of pathogenicity proteins in the network is 10.25, which is higher than that of the whole network (7.74). These pathogenicity genes are from the PHI-base website and a recent published paper (Jeon *et al.,* 2007). There are only common names and protein sequences available in the PHI-base. To get the standard *M. grisea* protein ID (according to the latest release of BROAD), a BLAST searching was employed. The identity between the sequence in PHI-base and that in the latest release of BROAD is shown in the table. ^b^ The Pfam domain annotations of those proteins with *E*-value < 0.01 are provided. The name and brief description of the PFAM domain and the number of this PFAM domain it contains are provided. The Pfam domain annotation was carried out by using Hmmer-2.3.2 and the Pfam database (Pfam_ls, release 22.0).

**Table S3 The 105 secreted proteins in the network. ^a,b,c^**

| **Protein** | **Degree** | **PFAM domain** | **Description** | **E value** | **Number** |
| --- | --- | --- | --- | --- | --- |
| MGG_00140 | 1 | PF06682 | Protein of unknown function (DUF1183) | 8.70E-08 | 1 |
| MGG_00488 | 1 | No hit. | - | - | - |
| MGG_00786* | 1 | No hit. | - | - | - |
| MGG_02134 | 1 | PF01019 | Gamma-glutamyltranspeptidase | 1.70E-199 | 1 |
| MGG_02630 | 1 | PF08739 | pH-response regulator, PalI / Rim9 | 2.00E-71 | 1 |
| MGG_02867 | 1 | PF01011 | PQQ enzyme repeat | 3.80E-06 | 2 |
| MGG_03197* | 1 | PF08551 | Eukaryotic integral membrane protein (DUF1751) | 5.10E-42 | 1 |
| MGG_03276 | 1 | No hit. | - | - | - |
| MGG_03476 | 1 | PF01135 | Protein-L-isoaspartate(D-aspartate) O-methyltransferase (PCMT) | 4.90E-47 | 1 |
| MGG_03634* | 1 | PF01545 | Cation efflux family | 3.50E-58 | 1 |
| MGG_03943* | 1 | PF00036 | EF hand | 9.80E-07 | 2 |
| MGG_04900 | 1 | PF00245 | Alkaline phosphatase | 3.30E-41 | 1 |
| MGG_05445* | 1 | PF00702 | haloacid dehalogenase-like hydrolase | 7.40E-18 | 1 |
| MGG_05832 | 1 | No hit. | - | - | - |
| MGG_06189 | 1 | No hit. | - | - | - |
| MGG_06303* | 1 | PF07859 | alpha/beta hydrolase fold | 4.10E-20 | 1 |
| MGG_06844 | 1 | PF01161 | Phosphatidylethanolamine-binding protein | 1.20E-16 | 1 |
| MGG_07068 | 1 | PF04193 | PQ loop repeat | 2.10E-26 | 2 |
| MGG_07234 | 1 | PF00254 | FKBP-type peptidyl-prolyl cis-trans isomerase | 6.10E-48 | 1 |
| MGG_07502* | 1 | PF01556 | DnaJ C terminal region | 0.0056 | 1 |
| MGG_08175* | 1 | No hit. | - | - | - |
| MGG_08319 | 1 | PF04389 | Peptidase family M28 | 8.00E-56 | 1 |
| MGG_08772 | 1 | PF01161 | Phosphatidylethanolamine-binding protein | 1.20E-07 | 1 |
| MGG_09465 | 1 | No hit. | - | - | - |
| MGG_10452 | 1 | No hit. | - | - | - |
| MGG_11149 | 1 | PF03856 | Beta-glucosidase (SUN family) | 6.90E-08 | 1 |
| MGG_11236 | 1 | PF00571 | CBS domain pair | 8.30E-08 | 1 |
| MGG_11913* | 1 | No hit. | - | - | - |
| MGG_12474 | 1 | PF00149 | Calcineurin-like phosphoesterase | 5.40E-15 | 1 |
| MGG_12799 | 1 | PF00082 | Subtilase family | 1.70E-39 | 1 |
| MGG_13557 | 1 | No hit. | - | - | - |
| MGG_13765 | 1 | PF05577 | Serine carboxypeptidase S28 | 1.90E-27 | 1 |
| MGG_14302* | 1 | PF08022 | FAD-binding domain | 7.00E-05 | 1 |
| MGG_00469 | 2 | PF07738 | Sad1 / UNC-like C-terminal | 6.20E-51 | 1 |
| MGG_02130 | 2 | PF07859 | alpha/beta hydrolase fold | 0.0032 | 1 |
| MGG_02275 | 2 | PF05577 | Serine carboxypeptidase S28 | 4.50E-10 | 1 |
| MGG_02557 | 2 | No hit. | - | - | - |
| MGG_03337 | 2 | PF05577 | Serine carboxypeptidase S28 | 5.70E-10 | 1 |
| MGG_03346 | 2 | PF01112 | Asparaginase | 2.00E-93 | 1 |
| MGG_03510* | 2 | PF06427 | UDP-glucose:Glycoprotein Glucosyltransferase | 1.90E-58 | 1 |
| MGG_03817 | 2 | No hit. | - | - | - |
| MGG_04045 | 2 | PF03200 | Mannosyl oligosaccharide glucosidase | 2.20E-253 | 1 |
| MGG_04507 | 2 | PF04628 | Sedlin, N-terminal conserved region | 7.60E-06 | 1 |
| MGG_04825 | 2 | No hit. | - | - | - |
| MGG_05489 | 2 | PF03639 | Glycosyl hydrolase family 81 | 3.20E-171 | 1 |
| MGG_05865 | 2 | PF09118 | Domain of unknown function (DUF1929) | 8.70E-24 | 1 |
| MGG_05989 | 2 | PF00326 | Prolyl oligopeptidase family | 2.50E-33 | 1 |
| MGG_06773 | 2 | No hit. | - | - | - |
| MGG_06906 | 2 | PF00246 | Zinc carboxypeptidase | 8.60E-100 | 1 |
| MGG_08305 | 2 | PF00328 | Histidine acid phosphatase | 1.40E-11 | 1 |
| MGG_08966 | 2 | PF05922 | Subtilisin N-terminal Region | 5.60E-12 | 1 |
| MGG_08994 | 2 | PF00857 | Isochorismatase family | 1.30E-06 | 1 |
| MGG_09692 | 2 | PF02225 | PA domain | 1.40E-15 | 1 |
| MGG_09918 | 2 | PF00722 | Glycosyl hydrolases family 16 | 3.00E-65 | 1 |
| MGG_09922 | 2 | PF02838 | Glycosyl hydrolase family 20, domain 2 | 4.60E-05 | 1 |
| MGG_10171 | 2 | PF01546 | Peptidase family M20/M25/M40 | 0.0042 | 1 |
| MGG_10533 | 2 | PF00491 | Arginase family | 2.00E-47 | 1 |
| MGG_10799 | 2 | PF00328 | Histidine acid phosphatase | 6.10E-15 | 1 |
| MGG_12467* | 2 | No hit. | - | - | - |
| MGG_13574* | 2 | PF03901 | Alg9-like mannosyltransferase family | 7.20E-07 | 1 |
| MGG_13626 | 2 | PF02065 | Melibiase | 4.10E-14 | 1 |
| MGG_01350 | 3 | No hit. | - | - | - |
| MGG_01692 | 3 | PF00194 | Eukaryotic-type carbonic anhydrase | 7.60E-07 | 1 |
| MGG_01858 | 3 | No hit. | - | - | - |
| MGG_04162* | 3 | PF00230 | Major intrinsic protein | 7.50E-41 | 1 |
| MGG_04527* | 3 | PF04080 | Per1-like | 1.80E-66 | 1 |
| MGG_04973 | 3 | PF00194 | Eukaryotic-type carbonic anhydrase | 1.70E-07 | 1 |
| MGG_06004* | 3 | PF01145 | SPFH domain / Band 7 family | 5.50E-33 | 1 |
| MGG_06060* | 3 | PF02535 | ZIP Zinc transporter | 4.60E-51 | 1 |
| MGG_06080* | 3 | PF01018 | GTP1/OBG | 2.20E-26 | 1 |
| MGG_07331 | 3 | PF00150 | Cellulase (glycosyl hydrolase family 5) | 0.0059 | 1 |
| MGG_07877 | 3 | PF00326 | Prolyl oligopeptidase family | 5.70E-50 | 1 |
| MGG_08647 | 3 | PF04886 | PT repeat | 2.70E-16 | 3 |
| MGG_10400 | 3 | No hit. | - | - | - |
| MGG_12317 | 3 | No hit. | - | - | - |
| MGG_00506* | 4 | PF02012 | BNR/Asp-box repeat | 5.40E-28 | 10 |
| MGG_05753* | 5 | PF01216 | Calsequestrin | 5.30E-05 | 1 |
| MGG_06175 | 5 | PF00085 | Thioredoxin | 1.80E-27 | 1 |
| MGG_13508 | 5 | PF08238 | Sel1 repeat | 5.60E-65 | 10 |
| MGG_01607* | 6 | PF00262 | Calreticulin family | 4.30E-204 | 1 |
| MGG_04500* | 6 | PF04756 | OST3 / OST6 family | 8.00E-31 | 1 |
| MGG_06336* | 6 | PF04142 | Nucleotide-sugar transporter | 0.0008 | 1 |
| MGG_07287 | 6 | PF01735 | Lysophospholipase catalytic domain | 2.20E-229 | 1 |
| MGG_08096 | 6 | PF01105 | emp24/gp25L/p24 family/GOLD | 5.30E-10 | 1 |
| MGG_00667 | 7 | PF01532 | Glycosyl hydrolase family 47 | 2.60E-123 | 1 |
| MGG_04086 | 7 | PF07646 | Kelch motif | 2.10E-17 | 5 |
| MGG_07807 | 7 | PF00188 | SCP-like extracellular protein | 1.20E-24 | 1 |
| MGG_08210* | 7 | No hit. | - | - | - |
| MGG_03620* | 8 | PF00083 | Sugar (and other) transporter | 2.80E-96 | 1 |
| MGG_03759 | 8 | PF04113 | Gpi16 subunit, GPI transamidase component | 7.80E-171 | 1 |
| MGG_03670 | 10 | PF05922 | Subtilisin N-terminal Region | 4.10E-30 | 1 |
| MGG_09271 | 10 | No hit. | - | - | - |
| MGG_03529* | 12 | No hit. | - | - | - |
| MGG_02503 | 13 | PF06723 | MreB/Mbl protein | 8.10E-06 | 1 |
| MGG_06574* | 13 | No hit. | - | - | - |
| MGG_09287 | 13 | PF04597 | Ribophorin I | 1.60E-144 | 1 |
| MGG_05097 | 15 | PF03694 | Erg28 like protein | 4.20E-20 | 1 |
| MGG_03687 | 16 | No hit. | - | - | - |
| MGG_07434* | 16 | PF02990 | Endomembrane protein 70 | 6.00E-243 | 1 |
| MGG_07124 | 18 | PF01336 | OB-fold nucleic acid binding domain | 9.60E-07 | 1 |
| MGG_02156 | 23 | PF00394 | Multicopper oxidase | 1.70E-46 | 1 |
| MGG_02821 | 25 | PF03345 | Dolichyl-diphosphooligosaccharide-protein glycosyltransferase 48kD subunit | 2.20E-100 | 1 |
| MGG_06722 | 27 | PF03198 | Glycolipid anchored surface protein (GAS1) | 4.10E-157 | 1 |
| MGG_04439 | 34 | PF01105 | emp24/gp25L/p24 family/GOLD | 2.20E-17 | 1 |
| MGG_10490 | 36 | PF01650 | Peptidase C13 family | 5.30E-15 | 1 |

^a^ We used Signalp3.0 to predict the secreted proteins in *M. grisea.* We chose the proteins which consistently predicted to be secreted by both the Neural Network (NN) and HMM methods. There are totally 105 secreted proteins in the network. The average degree is only 4.81, which is smaller than that of the whole network. ^b^ The Pfam domain annotations of those proteins with a *E*-value threshold of 0.01 are provided. The name and brief description of the PFAM domain and the number of this PFAM domain it contains are provided. The Pfam domain annotation was carried out by using Hmmer-2.3.2 and the Pfam database (Pfam_ls, release 22.0). **^c^** We utilized WoLFPSORT to predict the sub-cellular localization of these 105 *M. grisea* proteins. 28 proteins were not predicted to localize outside of the cell, which may be secreted into the rice cell and interact with rice proteins. These corresponding entries of these 28 proteins are labeled with asterisks.

**Table S4. The GO annotations of the 28 secreted proteins that may interact with rice protein.**^a^

| **Portein** | **GO term** | **GO category** | **GO accession number** |
| --- | --- | --- | --- |
| MGG_01607 | mycelium development | biological_process | GO:0043581 |
|  | protein metabolic process | biological_process | GO:0019538 |
|  | cellular macromolecule metabolic process | biological_process | GO:0044260 |
|  | intracellular membrane-bounded organelle | cellular_component | GO:0043231 |
|  | cytoplasm | cellular_component | GO:0005737 |
|  | cytoplasmic part | cellular_component | GO:0044444 |
|  | intrinsic to membrane | cellular_component | GO:0031224 |
|  | metal ion binding | molecular_function | GO:0046872 |
|  | cation binding | molecular_function | GO:0043169 |
|  | sugar binding | molecular_function | GO:0005529 |
| MGG_04527 | cellular lipid metabolic process | biological_process | GO:0044255 |
|  | protein metabolic process | biological_process | GO:0019538 |
|  | cellular macromolecule metabolic process | biological_process | GO:0044260 |
|  | biopolymer metabolic process | biological_process | GO:0043283 |
|  | macromolecule biosynthetic process | biological_process | GO:0009059 |
|  | lipid biosynthetic process | biological_process | GO:0008610 |
|  | lipid metabolic process | biological_process | GO:0006629 |
|  | cellular chemical homeostasis | biological_process | GO:0055082 |
|  | homeostatic process | biological_process | GO:0042592 |
|  | intracellular membrane-bounded organelle | cellular_component | GO:0043231 |
|  | endoplasmic reticulum part | cellular_component | GO:0044432 |
|  | endoplasmic reticulum membrane | cellular_component | GO:0005789 |
|  | cytoplasm | cellular_component | GO:0005737 |
|  | vacuolar membrane | cellular_component | GO:0005774 |
|  | cytoplasmic part | cellular_component | GO:0044444 |
|  | vacuolar part | cellular_component | GO:0044437 |
|  | endomembrane system | cellular_component | GO:0012505 |
|  | nuclear envelope-endoplasmic reticulum network | cellular_component | GO:0042175 |
| MGG_06336 | symbiosis, encompassing mutualism through parasitism | biological_process | GO:0044403 |
| MGG_12467 | intracellular membrane-bounded organelle | cellular_component | GO:0043231 |
|  | cytoplasm | cellular_component | GO:0005737 |
|  | cytoplasmic part | cellular_component | GO:0044444 |
|  | intrinsic to membrane | cellular_component | GO:0031224 |
|  | oxidoreductase activity, acting on heme group of donors | molecular_function | GO:0016675 |
|  | substrate-specific transmembrane transporter activity | molecular_function | GO:0022891 |
|  | heme-copper terminal oxidase activity | molecular_function | GO:0015002 |
| MGG_08175 | NO annotation. |  |  |
| MGG_14302 | intrinsic to membrane | cellular_component | GO:0031224 |
|  | metal ion binding | molecular_function | GO:0046872 |
|  | coenzyme binding | molecular_function | GO:0050662 |
|  | cation binding | molecular_function | GO:0043169 |
|  | purine nucleotide binding | molecular_function | GO:0017076 |
| MGG_06080 | purine nucleotide binding | molecular_function | GO:0017076 |
|  | ribonucleotide binding | molecular_function | GO:0032553 |
| MGG_07434 | intrinsic to membrane | cellular_component | GO:0031224 |
| MGG_03943 | metal ion binding | molecular_function | GO:0046872 |
|  | cation binding | molecular_function | GO:0043169 |
| MGG_00786 | protein localization | biological_process | GO:0008104 |
|  | secretion by cell | biological_process | GO:0032940 |
|  | protein secretion | biological_process | GO:0009306 |
|  | symbiosis, encompassing mutualism through parasitism | biological_process | GO:0044403 |
|  | intracellular membrane-bounded organelle | cellular_component | GO:0043231 |
|  | plasma membrane | cellular_component | GO:0005886 |
|  | cytoplasm | cellular_component | GO:0005737 |
|  | cytoplasmic part | cellular_component | GO:0044444 |
|  | intrinsic to membrane | cellular_component | GO:0031224 |
| MGG_05445 | symbiosis, encompassing mutualism through parasitism | biological_process | GO:0044403 |
|  | ion transport | biological_process | GO:0006811 |
|  | intrinsic to membrane | cellular_component | GO:0031224 |
|  | active transmembrane transporter activity | molecular_function | GO:0022804 |
|  | substrate-specific transmembrane transporter activity | molecular_function | GO:0022891 |
|  | metal ion binding | molecular_function | GO:0046872 |
|  | hydrolase activity, acting on acid anhydrides | molecular_function | GO:0016817 |
|  | purine nucleotide binding | molecular_function | GO:0017076 |
|  | ribonucleotide binding | molecular_function | GO:0032553 |
| MGG_04162 | intrinsic to membrane | cellular_component | GO:0031224 |
| MGG_03529 | intracellular membrane-bounded organelle | cellular_component | GO:0043231 |
|  | cytoplasm | cellular_component | GO:0005737 |
|  | cytoplasmic part | cellular_component | GO:0044444 |
| MGG_03197 | endomembrane system | cellular_component | GO:0012505 |
| MGG_04500 | transferase activity | molecular_function | GO:0016740* |
| MGG_03634 | ion transport | biological_process | GO:0006811 |
|  | substrate-specific transmembrane transporter activity | molecular_function | GO:0022891 |
| MGG_00506 | intrinsic to membrane | cellular_component | GO:0031224 |
| MGG_13574 | transferase activity, transferring glycosyl groups | molecular_function | GO:0016757 |
| MGG_06303 | No annotation. |  |  |
| MGG_07502 | protein metabolic process | biological_process | GO:0019538 |
|  | cellular macromolecule metabolic process | biological_process | GO:0044260 |
|  | unfolded protein binding | molecular_function | GO:0051082 |
|  | heat shock protein binding | molecular_function | GO:0031072 |
| MGG_06060 | ion transport | biological_process | GO:0006811 |
|  | substrate-specific transmembrane transporter activity | molecular_function | GO:0022891 |
| MGG_06574 | secretory pathway | biological_process | GO:0045045 |
|  | protein localization | biological_process | GO:0008104 |
|  | intracellular transport | biological_process | GO:0046907 |
|  | protein transport | biological_process | GO:0015031 |
|  | organelle organization and biogenesis | biological_process | GO:0006996 |
|  | mycelium development | biological_process | GO:0043581 |
|  | secretion by cell | biological_process | GO:0032940 |
|  | vesicle-mediated transport | biological_process | GO:0016192 |
|  | intrinsic to organelle membrane | cellular_component | GO:0031300 |
|  | intracellular membrane-bounded organelle | cellular_component | GO:0043231 |
|  | cytoplasm | cellular_component | GO:0005737 |
|  | cytoplasmic part | cellular_component | GO:0044444 |
|  | Golgi apparatus part | cellular_component | GO:0044431 |
|  | endomembrane system | cellular_component | GO:0012505 |
|  | Golgi membrane | cellular_component | GO:0000139 |
|  | intrinsic to membrane | cellular_component | GO:0031224 |
| MGG_03510 | biopolymer metabolic process | biological_process | GO:0043283 |
|  | macromolecule biosynthetic process | biological_process | GO:0009059 |
|  | protein metabolic process | biological_process | GO:0019538 |
|  | cellular macromolecule metabolic process | biological_process | GO:0044260 |
|  | transferase activity, transferring glycosyl groups | molecular_function | GO:0016757 |
| MGG_05753 | cell redox homeostasis | biological_process | GO:0045454 |
|  | homeostatic process | biological_process | GO:0042592 |
|  | intracellular membrane-bounded organelle | cellular_component | GO:0043231 |
|  | cytoplasm | cellular_component | GO:0005737 |
|  | cytoplasmic part | cellular_component | GO:0044444 |
|  | intramolecular oxidoreductase activity | molecular_function | GO:0016860 |
|  | disulfide oxidoreductase activity | molecular_function | GO:0015036 |
|  | electron carrier activity | molecular_function | GO:0009055 |
| MGG_08210 | secretory pathway | biological_process | GO:0045045 |
|  | protein localization | biological_process | GO:0008104 |
|  | secretion by cell | biological_process | GO:0032940 |
|  | vesicle-mediated transport | biological_process | GO:0016192 |
|  | intracellular transport | biological_process | GO:0046907 |
|  | protein transport | biological_process | GO:0015031 |
|  | intracellular membrane-bounded organelle | cellular_component | GO:0043231 |
|  | cytoplasm | cellular_component | GO:0005737 |
|  | cytoplasmic part | cellular_component | GO:0044444 |
|  | intrinsic to membrane | cellular_component | GO:0031224 |
| MGG_06004 | cell aging | biological_process | GO:0007569 |
|  | negative regulation of cellular metabolic process | biological_process | GO:0031324 |
|  | regulation of protein metabolic process | biological_process | GO:0051246 |
|  | regulation of cellular metabolic process | biological_process | GO:0031323 |
|  | negative regulation of protein metabolic process | biological_process | GO:0051248 |
|  | protein metabolic process | biological_process | GO:0019538 |
|  | cellular macromolecule metabolic process | biological_process | GO:0044260 |
|  | mitochondrial envelope | cellular_component | GO:0005740 |
|  | intracellular membrane-bounded organelle | cellular_component | GO:0043231 |
|  | mitochondrial membrane | cellular_component | GO:0031966 |
|  | cytoplasm | cellular_component | GO:0005737 |
|  | cytoplasmic part | cellular_component | GO:0044444 |
|  | mitochondrial part | cellular_component | GO:0044429 |
|  | organelle inner membrane | cellular_component | GO:0019866 |
| MGG_03620 | symbiosis, encompassing mutualism through parasitism | biological_process | GO:0044403 |
|  | carbohydrate transport | biological_process | GO:0008643 |
|  | intrinsic to membrane | cellular_component | GO:0031224 |
|  | active transmembrane transporter activity | molecular_function | GO:0022804 |
|  | substrate-specific transmembrane transporter activity | molecular_function | GO:0022891 |
| MGG_11913 | transferase activity | molecular_function | GO:0016740* |

^a^The GO terms at depth 4 or 3 in three categories are shown. For those GO terms at depth >4, their father nodes at depth 4 are listed. Meanwhile, we asterisked the GO terms at depth 3.
