# Supplementary material for: The prediction of protein-protein interaction networks in rice blast fungus
Source: BMC Genomics. 2008 Nov 2;9:519. doi: 10.1186/1471-2164-9-519 (PMC2601049; doi:10.1186/1471-2164-9-519)

Additional file 3. Clusters or communities containing pathogenicity genes (proteins). Those nodes represented by red triangles are pathogenicity genes. The description alongside each cluster is the enriched GO terms identified by Fisher exact test followed by FDR correction. The corresponding corrected *p*-value is also listed. This figure can be zoomed in to view the corresponding BROAD accession number of each node.

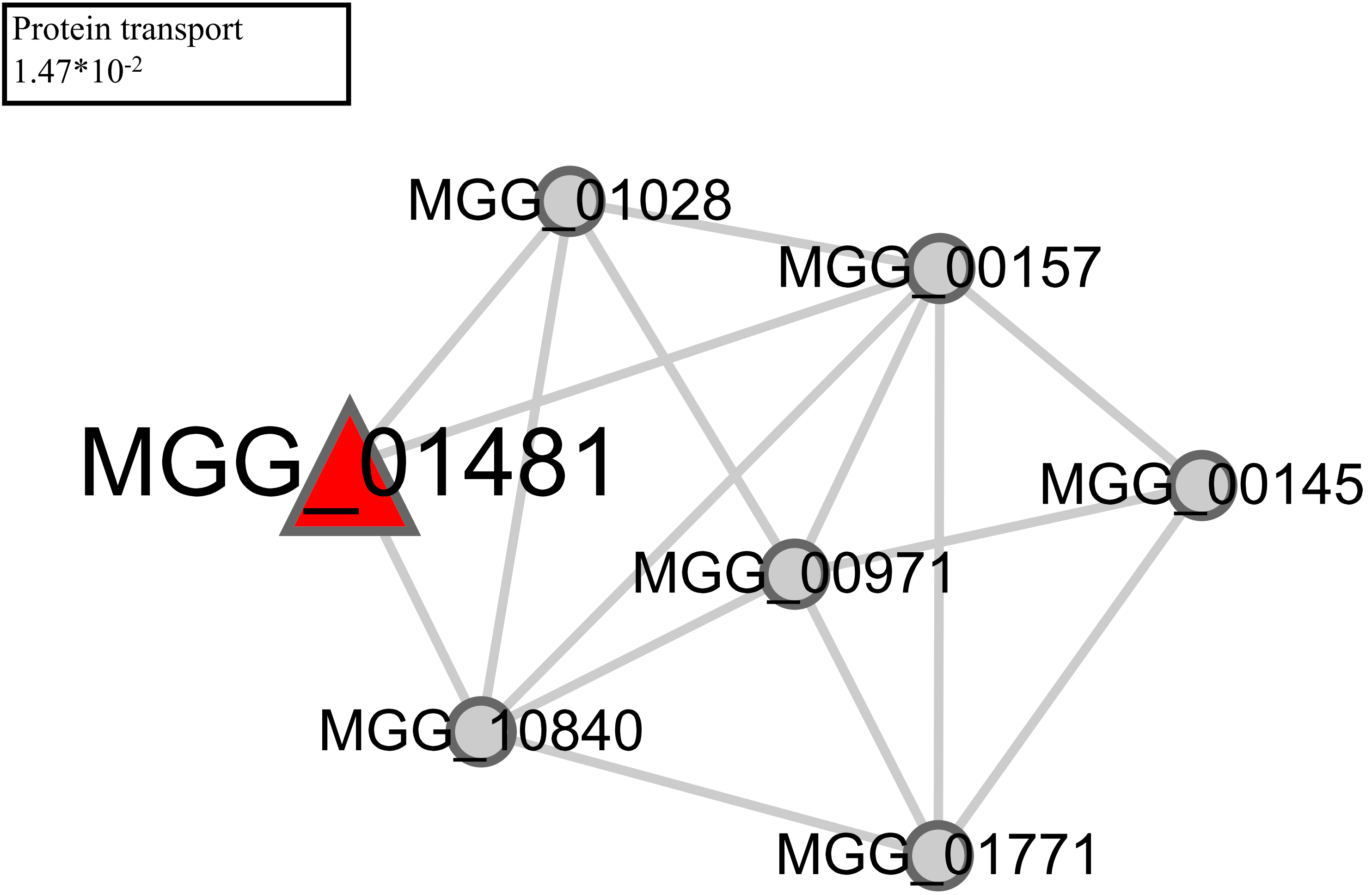

Protein transport
1.47*10-2
MGG_01028
MGG_00157
MGG_01481
MGG_00145
MGG_00971
MGG_10840
MGG_01771

Phosphorus metabolic process
$2.58*10^{-7}$

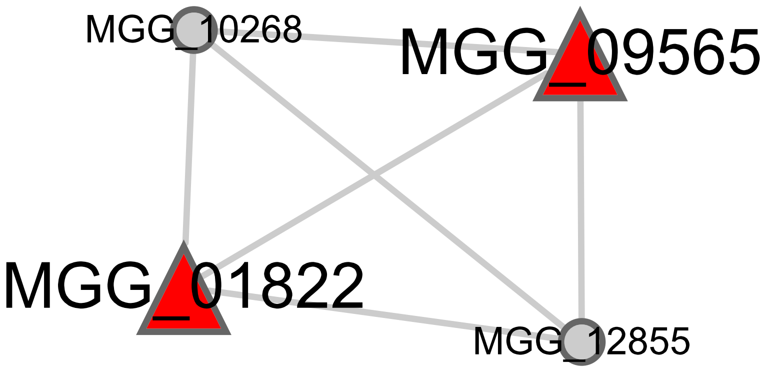

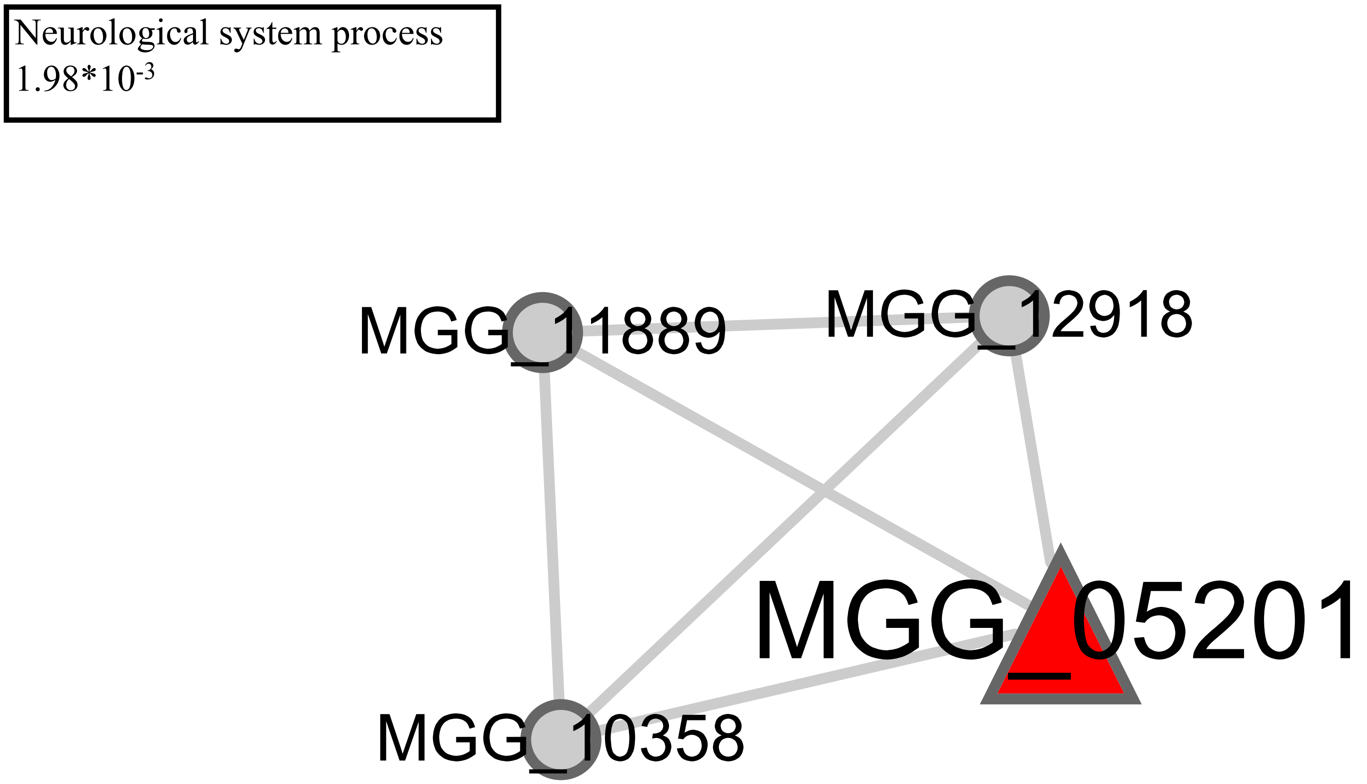
Neurological system process
1.98*10-3
MGG_11889
MGG_12918
MGG_05201
MGG_10358

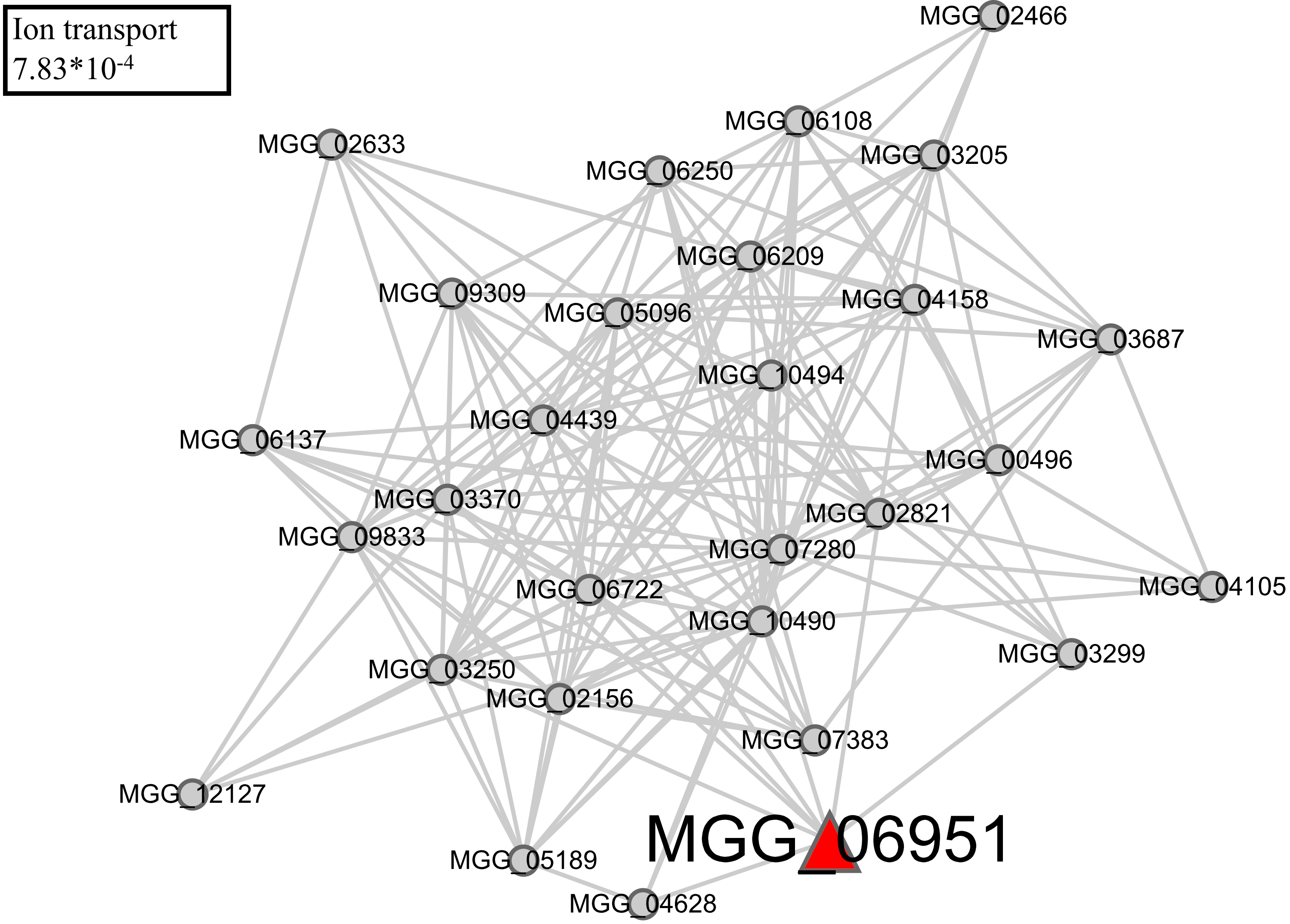
Ion transport
7.83*10-4
MGG_02466
MGG_06108
MGG_02633
MGG_03205
MGG_06250
MGG_06209
MGG_09309
MGG_04158
MGG_05096
MGG_03687
MGG_10494
MGG_04439
MGG_06137
MGG_00496
MGG_03370
MGG_02821
MGG_09833
MGG_07280
MGG_04105
MGG_06722
MGG_10490
MGG_03299
MGG_03250
MGG_02156
MGG_07383
MGG_12127
MGG_06951
MGG_05189
MGG_04628

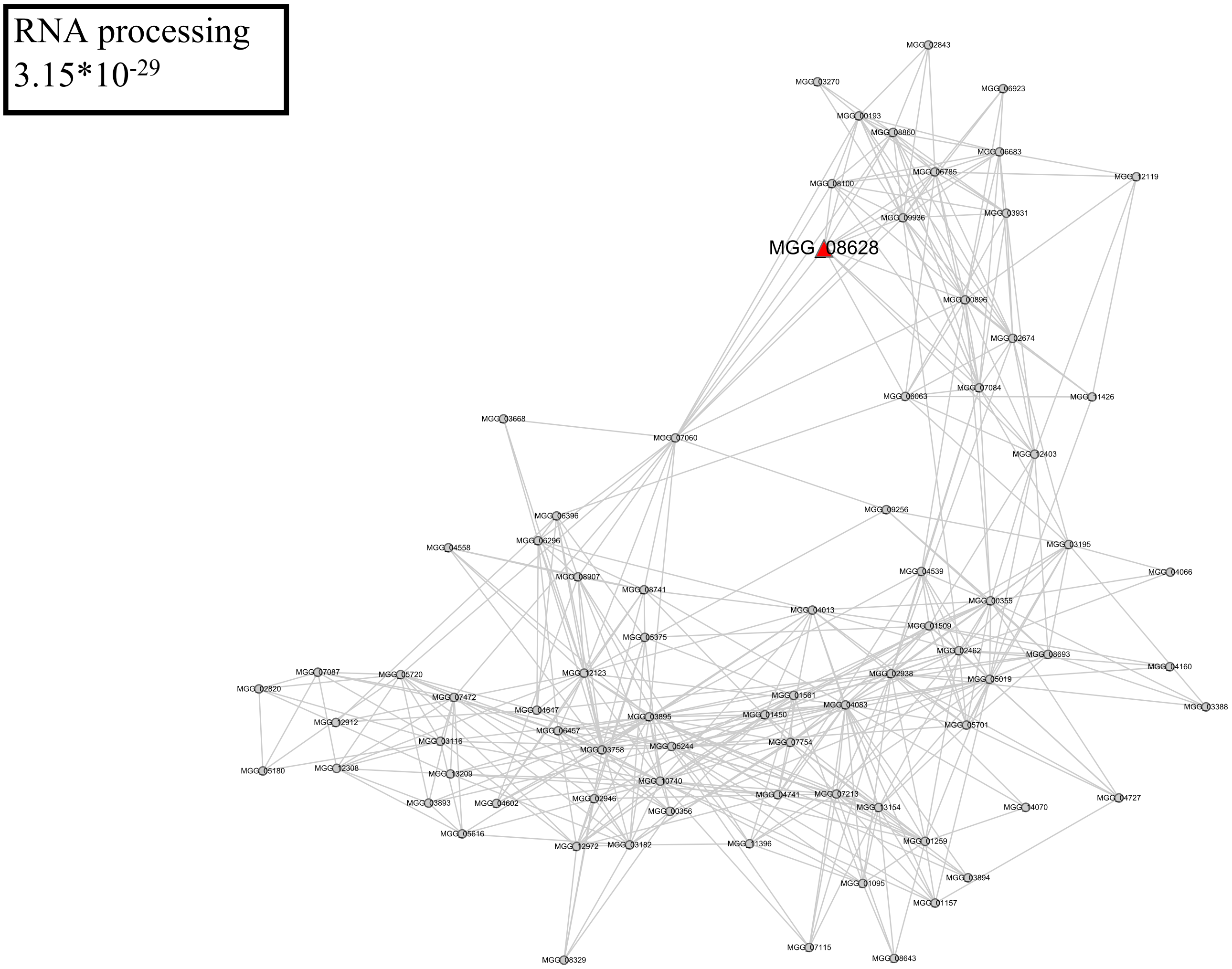
RNA processing
3.15*10-29
MGG_08628

Symbiosis, encompassing mutualism through parasitism
$1.75*10^{-2}$

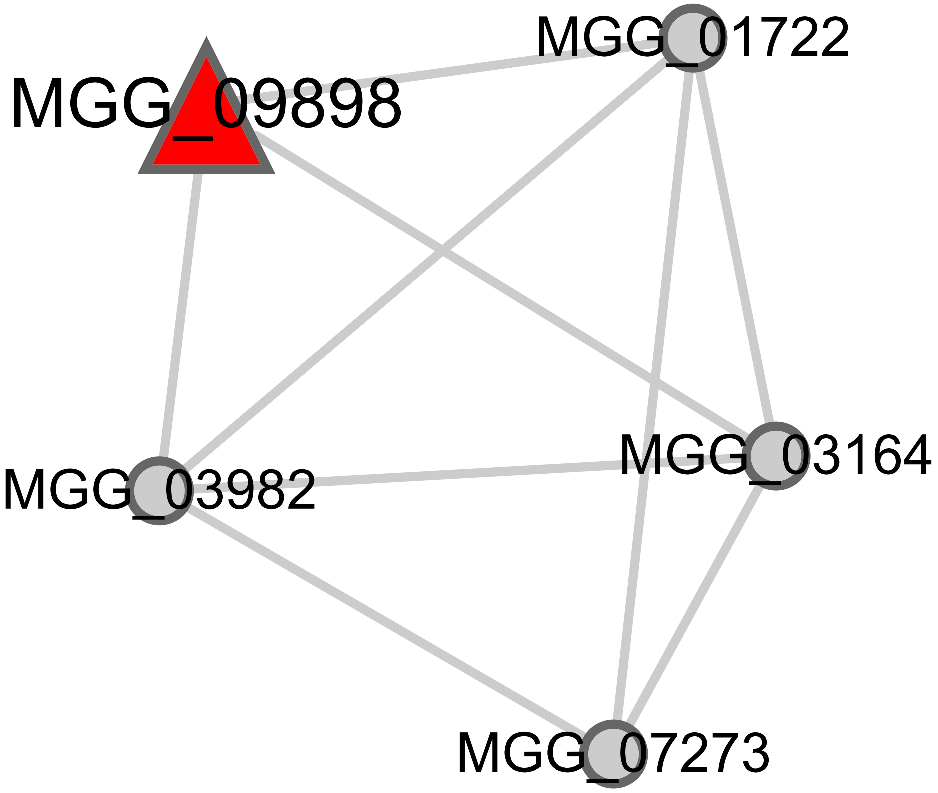

Chromatin silencing
$7.82*10^{-3}$

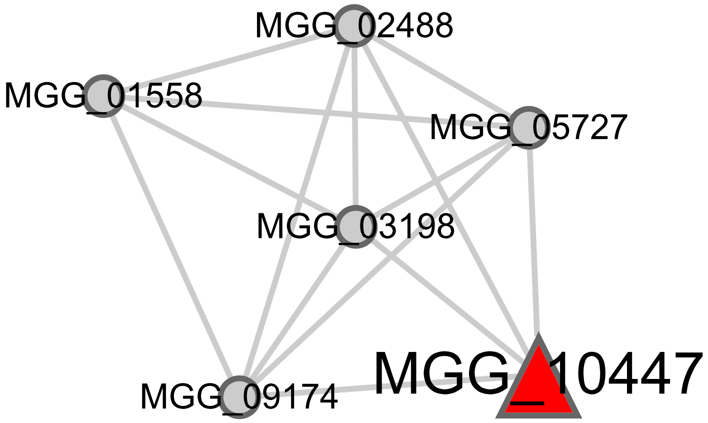

Supplement: Additional file 3 — Clusters or communities containing pathogenicity genes (proteins). This pdf file contains a larger version of Figure 3. [file 1471-2164-9-519-S3.pdf]
