# Supplementary material for: The prediction of protein-protein interaction networks in rice blast fungus
Source: BMC Genomics. 2008 Nov 2;9:519. doi: 10.1186/1471-2164-9-519 (PMC2601049; doi:10.1186/1471-2164-9-519)
Supplement: Additional file 4 — The network of pathogenicity proteins. This file contains a network graph showing 32 pathogenicity proteins and their interacting partners. [file 1471-2164-9-519-S4.pdf]

Additional file 4. The network of pathogenicity proteins.

This subnet consisted of the 32 pathogenicity proteins and their interaction partners. This figure can be zoomed in to view the corresponding BROAD accession number of each node. Triangle nodes indicated pathogenicity proteins, while the circular nodes denoted their interacting partners. The size of a circular node is proportional to its degree in the whole network. The golden triangle nodes represented pathogenicity genes uncovered by the high-throughput method, while the red triangle nodes stand for those collected from the PHI-base website.

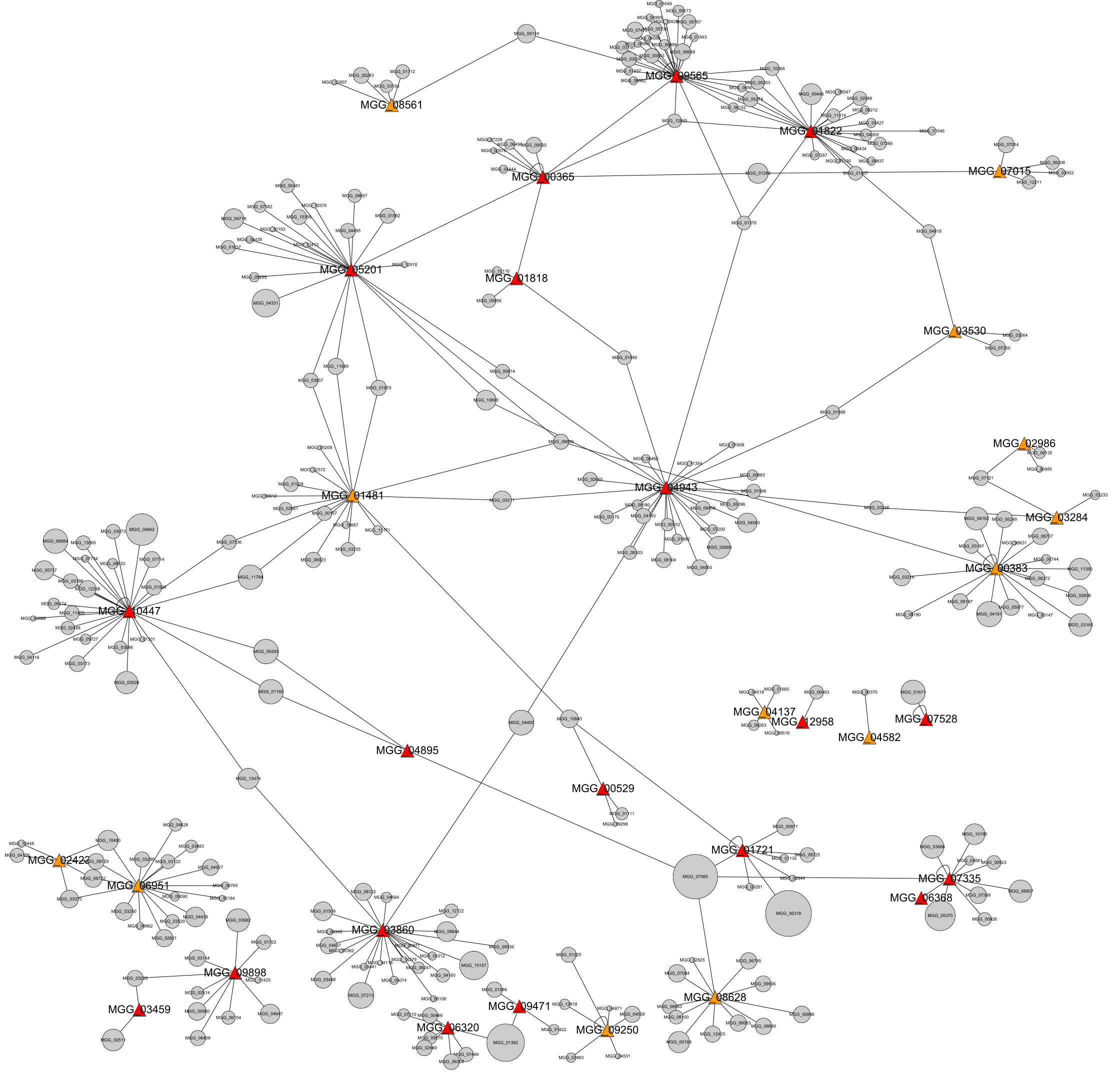
MGG_08561
MGG_09565
MGG_01822
MGG_00365
MGG_07015
MGG_05201
MGG_01818
MGG_03530
MGG_02986
MGG_04943
MGG_01481
MGG_03284
MGG_00383
MGG_10447
MGG_04137
MGG_12958
MGG_04582
MGG_07528
MGG_04895
MGG_00529
MGG_01721
MGG_07335
MGG_06368
MGG_02422
MGG_06951
MGG_03860
MGG_09898
MGG_03459
MGG_09471
MGG_06320
MGG_09250
MGG_08628
