# Supplementary material for: The prediction of protein-protein interaction networks in rice blast fungus
Source: BMC Genomics. 2008 Nov 2;9:519. doi: 10.1186/1471-2164-9-519 (PMC2601049; doi:10.1186/1471-2164-9-519)

Additional file 5. The network of secreted proteins.

This subnet consisted of 105 secreted proteins and their interaction partners. This figure can be zoomed in to view the corresponding BROAD accession number of each node. Diamond nodes are secreted proteins, while the circular nodes are their interacting partners. The size of a circular node is proportional to its degree in the whole network. Green diamond nodes are secreted proteins predicted to be secreted into the rice cell and the other secreted proteins are colored blue.

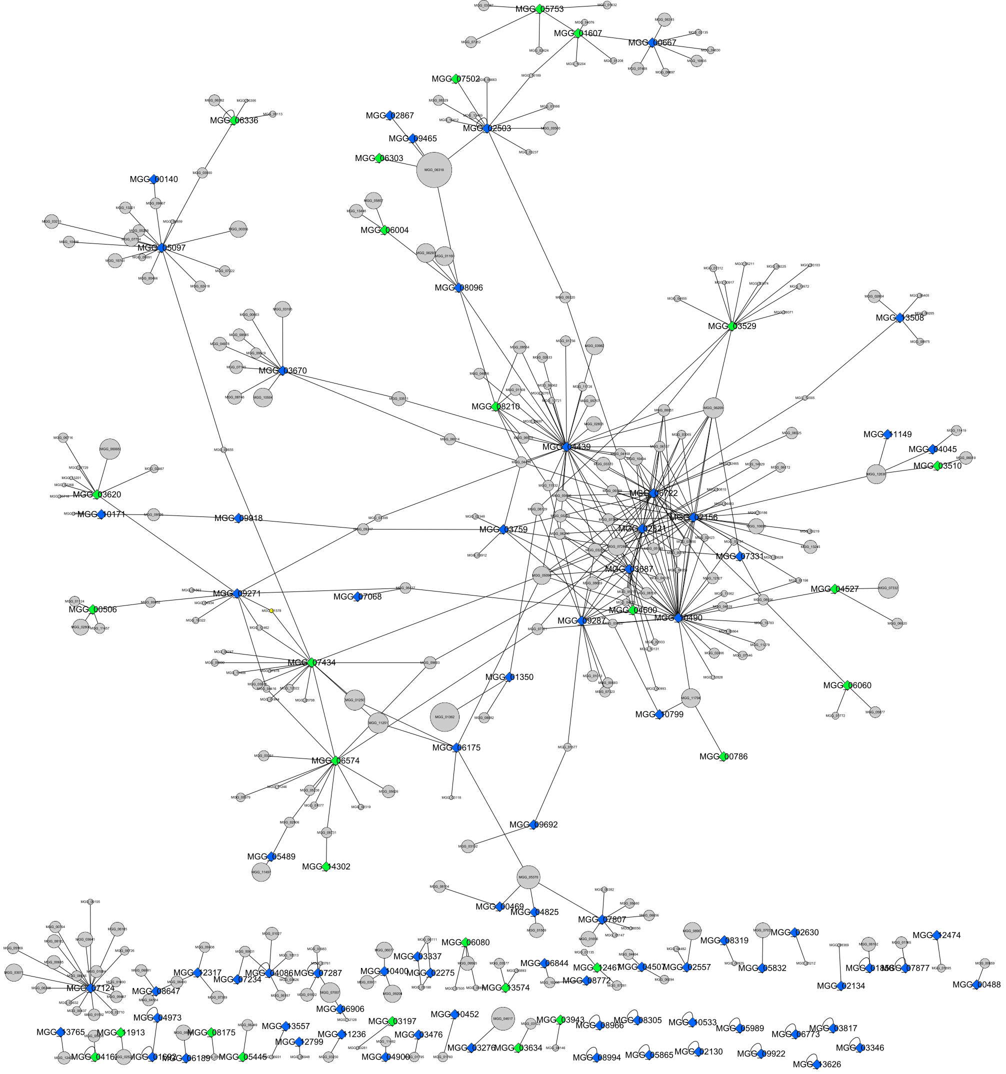

Supplement: Additional file 5 — The network of secreted proteins. This file contains a network graph showing 105 secreted proteins and their interaction partners. [file 1471-2164-9-519-S5.pdf]
