# Supplementary material for: The prediction of protein-protein interaction networks in rice blast fungus
Source: BMC Genomics. 2008 Nov 2;9:519. doi: 10.1186/1471-2164-9-519 (PMC2601049; doi:10.1186/1471-2164-9-519)
Supplement: Additional file 6 — The sources of proteome and PPI data of the model organisms. This file contains a table (i.e., Table S5) showing the websites and versions of the proteome and PPI data of the model organisms used in this paper. [file 1471-2164-9-519-S6.doc]

**Table S5. The sources of proteome and PPI data of other model organisms**

| Species | Proteome data | PPI data |
| --- | --- | --- |
| *S. cerevisae* | <ftp://genome-ftp.stanford.edu/pub/yeast/sequence/genomic_sequence/orf_protein/>  Query date: 2007.6  6719 sequences | [http://dip.doe-mbi.ucla.edu/dip](http://dip.doe-mbi.ucla.edu/dip/Download.cgi?SM=5)  version: Scere20080407  17579 interactions |
| *C. elegans* | <ftp://ftp.ncbi.nih.gov/genomes/Caenorhabditis_elegans/>  Query date: 2007.6  22844 sequences | [http://dip.doe-mbi.ucla.edu/dip](http://dip.doe-mbi.ucla.edu/dip/Download.cgi?SM=5)  version: Celeg20080407  4038 interactions |
| *D. melanogaster* | <ftp://ftp.ncbi.nih.gov/genomes/Drosophila_melanogaster/>  Query date: 2007.6  19765 sequences | [http://dip.doe-mbi.ucla.edu/dip](http://dip.doe-mbi.ucla.edu/dip/Download.cgi?SM=5)  version: Dmela20080407  22840 interactions |
| *E. coli* | <ftp://ftp.ncbi.nih.gov/genomes/Bacteria/Escherichia_coli_K12>  Query date: 2007.6  4131 sequences | [http://dip.doe-mbi.ucla.edu/dip](http://dip.doe-mbi.ucla.edu/dip/Download.cgi?SM=5)  version: Ecoli20080407  6976 interactions |
| *H. sapiens* | <http://www.hprd.org/sentDataRequest>  release 6  25205 sequences | <http://www.hprd.org/sentDataRequest>  version: release 7  37107 interactions |
